# Mass spectrometry imaging identifies altered hepatic lipid signatures during experimental *Leishmania donovani* infection

**DOI:** 10.3389/fimmu.2022.862104

**Published:** 2022-07-28

**Authors:** Roel Tans, Shoumit Dey, Nidhi Sharma Dey, Jian-Hua Cao, Prasanjit S. Paul, Grant Calder, Peter O’Toole, Paul M. Kaye, Ron M. A. Heeren

**Affiliations:** ^1^ Maastricht MultiModal Molecular Imaging (M4I) Institute, Division of Imaging Mass Spectrometry, Maastricht University, Maastricht, Netherlands; ^2^ York Biomedical Research Institute, Hull York Medical School, University of York, York, United Kingdom; ^3^ Department of Biology, University of York, York, United Kingdom

**Keywords:** leishmania, inflammation, granulomas, liver, lipids, mass spectrometry imaging, spatial analysis

## Abstract

**Introduction:**

Spatial analysis of lipids in inflammatory microenvironments is key to understand the pathogenesis of infectious disease. Granulomatous inflammation is a hallmark of leishmaniasis and changes in host and parasite lipid metabolism have been observed at the bulk tissue level in various infection models. Here, mass spectrometry imaging (MSI) is applied to spatially map hepatic lipid composition following infection with Leishmania *donovani*, an experimental mouse model of visceral leishmaniasis.

**Methods:**

Livers from naïve and *L. donovani*-infected C57BL/6 mice were harvested at 14- and 20-days post-infection (n=5 per time point). 12 µm transverse sections were cut and covered with norhamane, prior to lipid analysis using MALDI-MSI. MALDI-MSI was performed in negative mode on a Rapiflex (Bruker Daltonics) at 5 and 50 µm spatial resolution and data-dependent analysis (DDA) on an Orbitrap-Elite (Thermo-Scientific) at 50 µm spatial resolution for structural identification analysis of lipids.

**Results:**

Aberrant lipid abundances were observed in a heterogeneous distribution across infected mouse livers compared to naïve mouse liver. Distinctive localized correlated lipid masses were found in granulomas and surrounding parenchymal tissue. Structural identification revealed 40 different lipids common to naïve and d14/d20 infected mouse livers, whereas 15 identified lipids were only detected in infected mouse livers. For pathology-guided MSI imaging, we deduced lipids from manually annotated granulomatous and parenchyma regions of interests (ROIs), identifying 34 lipids that showed significantly different intensities between parenchyma and granulomas across all infected livers.

**Discussion:**

Our results identify specific lipids that spatially correlate to the major histopathological feature of *Leishmania donovani* infection in the liver, viz. hepatic granulomas. In addition, we identified a three-fold increase in the number of unique phosphatidylglycerols (PGs) in infected liver tissue and provide direct evidence that arachidonic acid-containing phospholipids are localized with hepatic granulomas. These phospholipids may serve as important precursors for downstream oxylipin generation with consequences for the regulation of the inflammatory cascade. This study provides the first description of the use of MSI to define spatial-temporal lipid changes at local sites of infection induced by *Leishmania donovani* in mice.

## Introduction

Lipids are key players in infection and inflammation, with a prominent role in host-pathogen interactions. Emerging studies have revealed that multiple lipid species regulate inflammatory responses both positively and negatively (1–3). For example, research on inositol phospholipids has shed light on the diverse roles of phosphatidylinositol 3-kinases in orchestrating specific aspects of innate and adaptive immune responses (1, 2). Lipid remodeling of glycerolipids, glycerophospholipids and prenols are associated with the activation and regulation of the innate immune response (4). Moreover, dynamic changes in glycerophospholipid composition have been observed during macrophage functional polarization in response to cytokines (5). In the context of infection, a variety of lipid species, including phospholipids (PLs), glycolipids (GLs) and cholesterol have been shown to influence the interactions of the host with clinically important viral, bacterial, protozoal and helminth pathogens, including HIV (6–8), *Mycobacterium tuberculosis* (6, 9), *Leishmania* spp (10–15). and *Schistosoma* (16–18). During infection, tissues undergo complex changes in cellularity and structure but there are few reports that address infection-associated changes in lipid metabolism in a spatially resolved manner. As with the study of gene and protein expression (19–23), overcoming this knowledge gap will likely be key to unravelling how changes to the lipidome impact on disease pathogenesis and immunity.

Matrix assisted laser desorption/ionization – mass spectrometry imaging (MALDI-MSI) is a widely used methodology for rapid visualization of spatially-resolved biomolecules in tissues (24, 25). A tissue surface is analyzed in a raster process from which, at a specific spatial resolution, mass spectra are generated for each analyzed pixel. This yields a molecular image that depicts the spatial distribution of proteins, lipids or metabolites that translates into a molecular image (26). Moreover, it promotes the integration of molecular images with gross tissue pathology (25, 27). The spatial analysis of molecules (*viz.* protein, lipids, or metabolites) using MSI offers great promise in different areas of biomedical research. For instance, MSI analysis of medulloblastomas yielded the identification of novel lipids that improved the understanding of metastatic processes (28). MSI has also been deployed for clinical decision making. For example, phenotypic characterization of intratumour heterogeneity revealed different molecular patterns that are associated with the survival rate of patients with gastric and breast carcinoma (29), whereas MALDI-MSI showed the capability of defining early ischemic kidney injury (30). Furthermore, MSI has also proved its added value in digital pathology. A recent study by Ščupáková et al. (31) reported a pathology-guided MSI workflow, where hematoxylin and eosin (H&E) stained images were co-registered with MS images, allowing biomolecular image annotation at the single cell level. However, the use of MSI to examine the pathogenesis of infectious disease has to date been poorly exploited.

Leishmaniasis is a globally important yet neglected vector-borne disease, endemic in 98 tropical and subtropical countries across Africa, Asia, southern Europe, and South and Central Americas (32–34). In the mammalian host, *Leishmania* amastigotes are obligate intracellular parasites residing in the phagolysosome of myeloid cells (11, 35) where they exist as dormant and replicative forms. Experimental models of leishmaniasis as well as clinical research has identified an important role for lipids and lipid metabolism, spanning processes involved in the host-pathogen interaction at the cellular level through to determination of disease outcome. For example, infection-induced alterations in host plasma membrane lipids/lipid rafts can reduce the ability of macrophages to form immunological synapses with T cells, hindering the acquisition of immunity, and lipid bodies have been reported around the phagolysosome (10, 35). Similarly, cholesterol depletion has been reported in human visceral leishmaniasis (36–38) and cholesterol supplementation can lead to enhanced cure in experimental models of this disease (39, 40). The formation of granulomas, a type of focal inflammation characterized by a predominantly mononuclear cell composition, is also a hallmark of many forms of leishmaniasis, providing a spatial context in which immune mechanisms operate to control and contain infection.

A recent study applied MSI to evaluate lipid metabolism in the cutaneous lesion caused by *L. mexicana* infection in susceptible BALB/c mice, describing altered lipid profiles in quiescent and replicative amastigotes in a spatially defined way (41). In contrast to the macroscopic skin lesion caused by *L. mexicana*, where multiple granulomas may coalesce with associated necrosis and other cellular infiltrates, the hepatic granulomatous response to *L. donovani* infection in both resistant and susceptible strains of mice provides for a more discrete identification of individual granulomas, each formed around an initiating infected Kupffer cell and embedded within the hepatic parenchyma (42, 43). Thus, hepatic granuloma formation in this infection model provides a unique opportunity to not only compare changes to lipid metabolism that occur in the granuloma microenvironment versus those that occur in the parenchyma, but also to compare lipid metabolism across multiple granulomas within individual mice, exploring granuloma evolution and heterogeneity. Exploiting this model, we used MALDI-MSI to evaluate the topological distribution of different lipids in the infected mouse liver and tandem MS for subsequent lipid identification. Thus, all samples were imaged by three different modalities, *viz.* two different MS imaging methodologies and H&E staining that allowed us to overlay spatial distributions of specific lipid classes with morphological characteristics of the infected livers. Our data demonstrate different spatially- resolved lipid compositions within hepatic granulomas and the surrounding parenchyma.

## Methods

All solvents were purchased from Biosolve Chimie SARL (Dieuze, France) unless stated otherwise. Eosin-Y (Avantor), Gill’s hematoxylin, tert-Butyl methyl ether anhydrous-99.8%, 2,6-Di-tert-butyl-4-methylphenol, and norharmane were purchased from Sigma-Aldrich (Zwijndrecht, The Netherlands). Super Frost (SF) glass slides were obtained from VWR, (Breda, The Netherlands). Indium Tin Oxide (ITO) glass slides were obtained from Delta Technologies (Loveland, USA).

### Animal tissue samples

Adult specific-pathogen-free (SPF) C57BL/6 mice (originally obtained from Charles River, UK) were bred and maintained at the University of York according to UK Home Office guidelines. Genetic profiling of mice from the colony using microsatellite markers was conducted at Surrey Diagnostics Ltd (Cranleigh, UK), confirming identity to C57BL/6J at 27 microsatellite markers. Two mice (of four tested) had an additional allele at marker 138 (192bp), one mouse had an additional allele at marker 134 (112bp) and two mice had an additional allele at marker 144 (195bp). Mice were kept in individual ventilated cages at 20–21°C and 56% humidity under SPF conditions (FELASA 67M and 51M) and provided with food and water ad libitum and with cage enrichment. Mice included in experiments were six-eight-week-old females, of excellent health status and that had not been subject to any genetic manipulation or previous regulated procedures. For *L. donovani* infections, mice were infected once with 3×10^7^ amastigotes of an Ethiopian strain of *L. donovani* (LV9) *via* the intravenous route without anesthesia, and infection allowed to proceed for 14 or 20 days. Mice were infected as a single cohort and animals selected randomly at the two time points studied. At day 14 and 20 p.i., mice (n=5 per time point) were killed by the Home Office approved method of CO_2_ inhalation followed by cervical dislocation. Livers were removed post mortem. The liver from five six-week-old naïve female C57BL/6J mice, which was kept in similar conditions as described above, were used as a baseline control.

**Figure 1:**
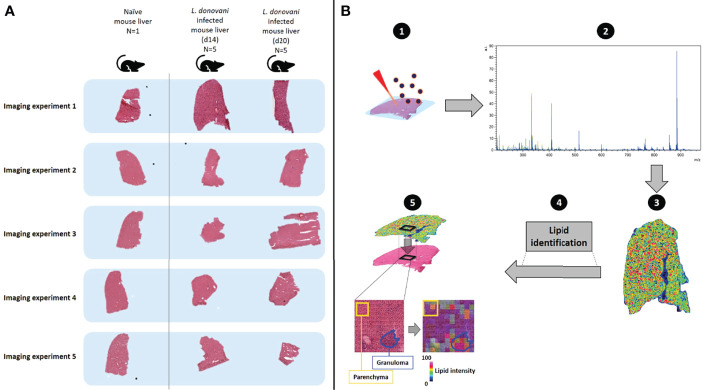
Schematic describing workflow for monitoring spatially-resolved lipids during hepatic *Leishmania donovani* infection. **(A)** Livers were obtained from a naïve C57BL/6 mouse and from *L. donovani*-infected C57BL/6 mice at d14 and d20 p.i. (n=5 mice per time point). Samples were sequentially measured, using the naïve tissue as a baseline and comparator (n=1). In addition, consistency of naïve mouse livers (n=5) is shown in **Figure S1**. **(B)** The spatial distribution of lipids and their respective structural identification was assessed by MALDI-MSI (1): a chemical matrix was applied to the tissue surface – this absorbs lipids from the tissue while retaining spatial localization. Subsequently, lipids were ionized by MALDI, which generates charged ions in the gas-phase (2). A mass analyzer is used to determine the mass-to-charge (*m/z*) ratio of these ions from which (3) the ions can be visualized to reveal their spatial localization and relative abundance across the analyzed tissue (4). Structural identification of spatially- resolved lipids was performed by tandem mass spectrometry (5). H&E-stained tissues were co-registered with mass spectrometry images, to allow integration of molecular lipid information from annotated regions of interest (*i.e.* granulomas and surrounding parenchyma) with histopathology.

Liver tissue from infected and control mice was snap frozen in liquid nitrogen and wrapped in aluminum foil at the University of York prior to shipment to Maastricht University. Subsequent analysis was conducted blind to sample origin. Cryo-embedded tissue sections were cut at a thickness of 12 μm using a CM1860 UV cryo-microtome at −20°C (Leica Microsystems, Wetzlar, Germany) and arranged on glass slides, each containing one naive, one d14 and one d20 section (see **Figure 1A** for experimental design). Slides were kept at -80°C until MALDI matrix application and further MSI analysis.

### Sample preparation

The MALDI matrix solution, norharmane (7 mg/ml in 2:1 chloroform: methanol), was applied using an automated TM sprayer (HTX Technologies, NC, U.S.A.) with the spray nozzle temperature at 30°C; number of passes, 3; flow rate, 0.12 ml/min; spray nozzle velocity, 1200 mm/min; track spacing, 3 mm; nitrogen gas pressure, 10 psi; drying time between passes, 30 s; allowing homogeneous matrix application.

H&E staining of samples was conducted post MALDI-MSI using an in-house protocol. In short, MALDI-matrix was removed by incubating the samples in 70% ethanol, followed by staining with 0.1% Gill’s hematoxylin for 3 minutes, staining with 0.2% eosin for 30 seconds, a short rinse in 70% ethanol, dehydrated in 100% ethanol for 2 minutes, equilibrated in xylene for 5 minutes, and mounted using a xylene based mounting medium and a glass cover slip. After the staining and drying, the slides were imaged with a microscope using bright field light microscope Leica Aperio CS2 slide scanner (Leica Microsystems, Nussloch, Germany).

### MALDI-MSI

Lipid MALDI-MSI was performed in negative ionization mode on a Bruker RapifleX (Bruker Daltonik, GmbH, Germany) and on a Thermo Orbitrap Elite mass spectrometer (Thermo Fisher Scientific, Bremen, Germany) equipped with an elevated-pressure MALDI ion source (Spectroglyph LLC, Kennewick, WA, USA), for subsequent lipid identification. In short, Bruker RapifleX MSI experiments were performed at 50 µm spatial resolution in reflector mode with 500 shots per pixel. Lipids were analyzed in the 200−1800 m/z range using red phosphorus as an internal calibrant.

Subsequent tandem MS for lipid identification was performed as described by Ellis et al., 2018 (44). To elaborate, MSI experiments were conducted at 25 µm (horizontal) and 50 µm (vertical) spatial resolution step-sizes. Subsequently, parallel full-scan FTMS (Orbitrap) and IT-MS/MS (ion trap) scans at adjacent 25-µm positions were conducted at a mass resolution of 240,000 and lipids were analyzed in the 200 – 1600 m/z range.

### Mass spectrometry imaging processing and lipid identification

FlexImaging v5.0 (Bruker Daltonik GmbH, Bremen, Germany) and LipostarMSI v1.1.0b28 (Molecular Horizon, Perugia, Italy) (45) were used to (pre)process the mass spectrometry imaging data acquired with the Bruker RapiFlex. Mass images, generated on the RapiFlex, were processed and visualized at a mass resolution of 15,000 with a 0.2 Da mass offset to show aberrant distribution and abundances of spatially resolved lipids. All imaging datasets were manually recalibrated using FlexImaging and FlexAnalysis (Bruker Daltonik GmbH, Bremen, Germany).

Data-dependent acquisition (DDA)-imaging, as described by Ellis et al. (2018) (44), was used for the structural lipid identification: raw full scan FTMS and IT-MS/MS spectra were generated using the Thermo Orbitrap Elite mass spectrometer. All spectra were recalibrated using an offline application called RecalOffline Application (Thermo Fisher Scientific, Bremen, Germany). Raw full scan FTMS mass images were processed and visualized at a high mass resolution of 240,000 and 0.0 Da mass offset. These (parent ion) masses were used for subsequent structural lipid identification using the corresponding generated IT-MS/MS spectra. All MS/MS data files were converted into imzML and further analyzed using LipostarMSI. Lipids were identified using the Lipid Maps database (edition July 2020) (46), incorporated into LipoStar MSI. Lipid searches, from the Lipid Maps database, were done for the [M-H]^-^ ion at a mass tolerance of 0.00 Da ± 3.00 ppm. MS/MS analyses were conducted on the precursor [M-H]^-^ ions and were matched with a *m/z* tolerance of 0.25 Da ± 0.00 ppm or lower. In addition, only lipids with a minimum carbon chain length of 12 were used for identification.

### Pathology-guided MSI

H&E-stained images of all mouse livers were processed with Qupath (0.2.3, Queen’s University Belfast; available at https://qupath.github.io/) (47). To execute a pathology-guided MSI analysis, 10 granulomas and 10 parenchymal regions were manually annotated as regions of interest (ROIs) on all *Leishmania* samples (n=5 mice per time point). Using an in-house built script for Qupath, coordinates of the annotated ROIs were exported and imported into LipostarMSI for subsequent co-registration of H&E images, annotated ROIs, and MS images. In addition to selection and importation, all ROIs were processed with alignment parameters set at MS tolerance of 0.00 Da. + 3.00 ppm and MS/MS tolerance of 0.25 Da. + 0.00 ppm. Lipid data was deduced from those pixels that fell within the rim of the manually drawn ROIs. In addition, lipids that were not identified in all ROIs were filtered out and not used for further analysis.

### Determination of parasite tissue distribution

Tissue sections adjacent to those used for MSI were used to determine the frequency of granulomas that contained visible parasites. Tissue was stained with Yoyo1 (ThermoFisher, Waltham, MA) to visualize host and parasite nuclei (AF488 channel) and antibody to *Leishmania* Oligopeptidase B (OpB; AF647 channel; provided by Jeremy Mottram, University of York, UK). Images were acquired using a Zeiss AXIOSCAN Z1 slide scanner (Carl Zeiss GmbH, Cambourne, UK) and imported into QuPath. Granulomas (44 -128 per section per mouse) were manually identified and scored as parasite positive or negative based on OpB staining.

### Data analysis

The multivariate analysis technique principal component analysis (PCA) was used to reduce the dimensionality of the spatial and mass resolution data per pixel allowing visualization of changes in lipid masses per spatial region, which allowed a more comprehensive visualization of multiple masses at once (48, 49). PCA and ANOVA fold changes analyses were performed using LipostarMSI. GraphPad Prism (v9.2.0, San Diego, USA) was used to generate heatmaps. P-values, for the ANOVA fold change analyses, were adjusted for multiple comparisons using the Benjamini–Hochberg procedure. A p-value lower or equal to 0.05 was considered significant. Raw MSI data were deposited on the METASPACE platform (50).

## Results

### Monitoring spatially resolved lipids in the liver during *L. donovani* infection

*L. donovani* infection in mice leads to the development of a potent granulomatous inflammatory response in the liver (51). To identify how changes in liver lipid composition develop in a spatially resolved way, we infected C57BL/6 mice with *L. donovani* and compared lipid composition in the hepatic parenchyma with that in granulomas at two time points during the early evolution of the granulomatous response. We analyzed five independent mice at d14 and d20 post-infection (p.i.) to allow both inter- and intra-mouse changes in lipid profile to be quantified. A liver from a control uninfected mouse was used to provide a baseline. A total of five consecutive slides from the control liver were sliced and combined (one by one) to one series of d14 and d20 infected mice, which allowed us to analyze the samples as five independent imaging experiments (**Figure 1A**). MALDI-MSI, MALDI-MS/MS and H&E staining were performed on each section to visualize and identify spatially-resolved lipids and allow co-registration of the molecular data to tissue histopathology (**Figure 1B**). Based on an analysis of adjacent tissue sections, the frequency of granulomas containing readily detectable parasites was 71 ± 11% at day 14 p.i. and 45 ± 12% at day 20 p.i. (p<0.01), in keeping with the expected kinetics of the host response in this mouse strain (42).

### Aberrant distribution and abundances of spatially resolved lipids in the *Leishmania*-infected liver

**Figure 2:**
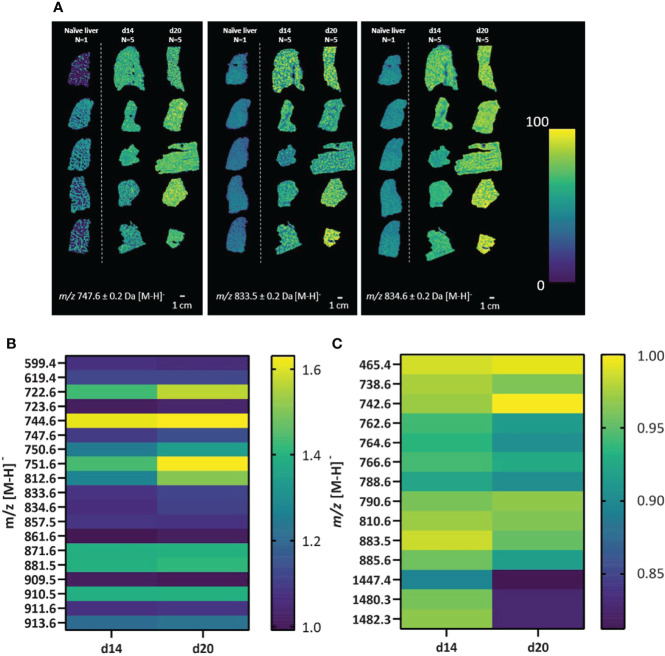
Alteration in lipid masses in naïve and *L. donovani*-infected mouse liver. Liver sections from *L. donovani-*infected C57BL/6 mice at d14 and d20 p.i. were compared to sections from naïve liver. **(A)** Spatial distributions of the relative abundance of three representative lipid masses (± 0.2 Da) in naïve, d14 and d20 livers. **(B, C)** Intensities of lipid masses increased in abundance **(B)** or decreased in abundance **(C)** in d14 (n=5) and d20 (n=5) mouse livers. Data are shown as relative mean fold change in infected vs naïve samples, as indicated in scale bar.

The spatial distribution of lipids was assessed across the full tissue area of all samples from the 5 imaging experiments depicted in **Figure 1A**. We first here aimed at evaluating whether lipid profiles are distinctively spatially resolved and show aberrant intensities between naïve and infected mouse livers. Using a fast lipid MALDI-MSI approach in negative mode at 50 x 50 µm spatial resolution, we observed a total of 33 (lipid) masses spatially-distributed across all tissues with heterogeneous abundances (**Figure 2**). For example, *m/z* 747.6 ± 0.2 Da, *m/z* 833.5 ± 0.2 Da and *m/z* 834.6 ± 0.2 Da had a higher intensity in samples from d14 and d20 infected mice compared to naïve comparator, but with clear heterogeneity in abundance across the tissue (**Figure 2A**). Interestingly, 19 lipid masses had higher overall intensities across all infected d14 and d20 livers (**Figure 2B**) whereas 13 lipid masses had lower overall intensities across all infected d14 and d20 livers compared to the naïve liver (**Figure 2C**). Hence, *L. donovani* infection is associated with marked alteration to the lipid composition of the liver. In addition, the spatial distribution of lipids was assessed across a total of five individual naïve mouse livers using a similar MALDI-MSI approach as described above to confirm consistency in lipid signals across naïve livers (Figure S1). Related to the lipid profiles observed from the naïve mouse liver in **Figures 1**, **2**, we observed overlapping mass spectra, and their respective intensities, across all naïve mouse livers (n=5). The naïve mouse livers (n=5) showed consistency in spatial distributions of lipids (**Figures S1B–F**), making the naïve liver (n=1) as shown in **Figures 1**, **2** a valid comparator.

### High-resolution MS imaging reveals distinctive localized correlated lipid masses in granulomas and surrounding parenchymal tissue

**Figure 3:**
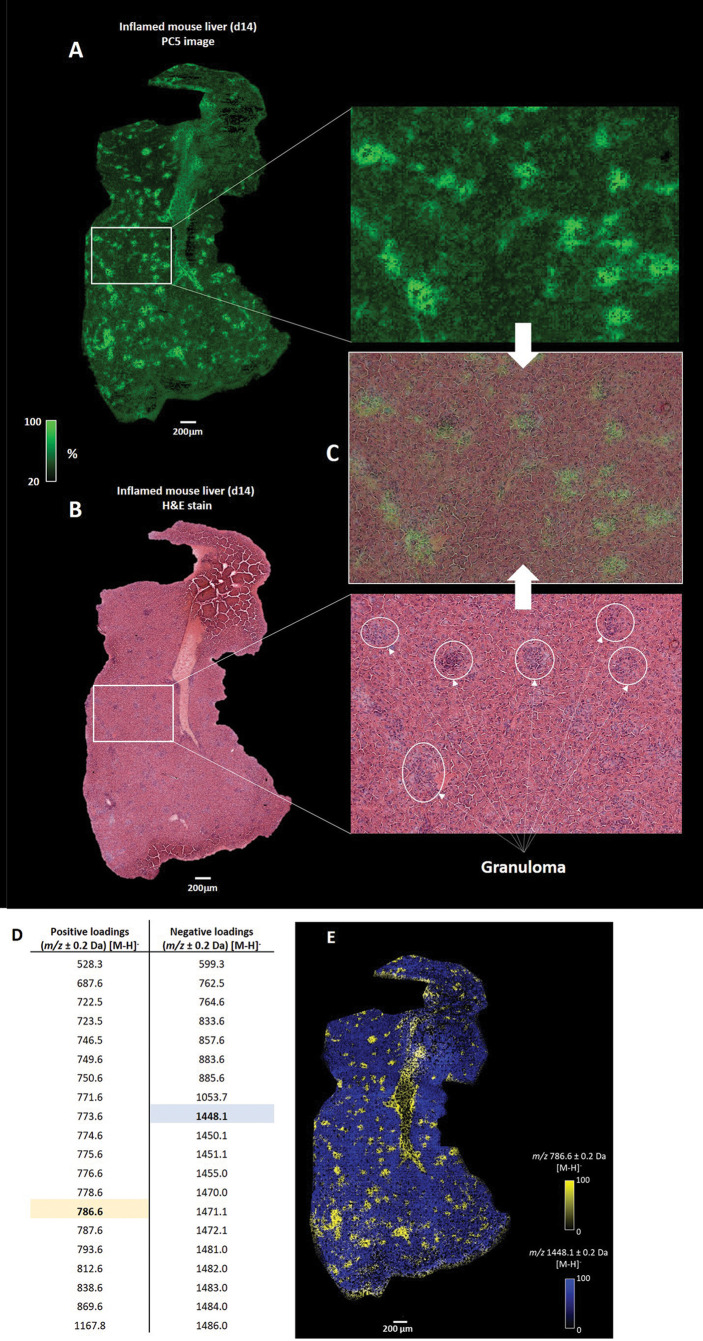
Integration of MSI and histopathology. **(A)** Principal component analysis (PCA) score image of the fifth principal component (PC5) shows the variance of correlated spatially-resolved lipid masses from a d14 *L. donovani* - infected mouse liver. **(B)** Post MALDI-MSI H&E staining of the tissue allows the histopathological annotation of granulomas. **(C)** Co-registration of the H&E-stained and the PC5 image reveals that lipid masses, correlated with the bright green regions, overlap with observed granulomas. Only a selection of granulomas are highlighted **(D)** Top 20 positive and negative loadings from the PC5 images, which represent the correlated lipid masses to the bright green (positive loadings) and dark green (negative loadings) regions. **(E)** Overlaid image of the spatial distribution of the relative abundance of *m/z* 786.6 (highlighted in yellow in **D**) and *m/z* 1448.1 (highlighted in blue in **D**).

To further define the heterogeneity in the distributions of lipid masses, we deployed high-resolution MSI, at a single cell level (5 µm pixel size), using a d14 *L. donovani* infected liver sample. To find patterns of spatially correlated lipid masses across the morphological characteristics of the tissue, we performed principal component analysis (PCA). Behind each principal component (PC), a list of unique masses, correlated for a specific region, was generated. The first four PCs discriminated chemical artifacts between on- and off-tissue (**Figure S2**) that were not correlated to the morphological characteristics of the tissue. In contrast, the PCA score image from PC5 discriminated on-tissue lipid masses that were spatially correlated in different regions (**Figure 3A**). Furthermore, tissue histopathology defined by H&E staining post MALDI-MSI (**Figure 3B**) was superimposable with the PC5 image, with granulomas clearly overlapping with the bright green regions generated in the PC5 image (**Figure 3C**).

Having identified that granulomas corresponded to specific regions within the PC5 image, we next deduced the top 20 positive and negative loadings that represent the unique lipid masses discriminative for the bright and dark green regions (i.e., granuloma and parenchyma, respectively) (**Figure 3D**). As an example, overlaying images of *m/z* 786.6 ± 0.2 Da and *m/z* 1448.1 ± 0.2 Da, deduced from the positive loadings and negative loadings respectively, showed unique spatial distributions (**Figure 3E**). We also observed similar spatial distributions for the other positive and negative loadings, of which several examples are provided (**Figure S3**). In addition, *m/z* values 786.6 ± 0.2 Da and 1448.1 ± 0.2 Da showed spatially-inversed intensities (**Figure S4**), which confirmed that *m/z* 786.6 and *m/z* 1448.1 were spatially resolved in distinct regions of the tissue.

### Structural identification of spatially-resolved lipids in *L. donovani-*infected mouse liver

In addition to the evaluation of spatially-resolved lipids across *L. donovani-*infected mouse livers as presented above, we combined high resolution imaging screening with structural identification of resolved lipids on the same samples as depicted in **Figures 1**, **2**. This approach allowed us to further scrutinize which specific spatially-resolved lipids were associated with infected mouse liver. In line with results depicted in **Figure 2**, we observed lipid masses with higher intense but heterogeneous distributions across all infected d14 and d20 samples, with d20 showing the highest intensity, compared to naïve mouse liver (data not shown).

**Figure 4:**
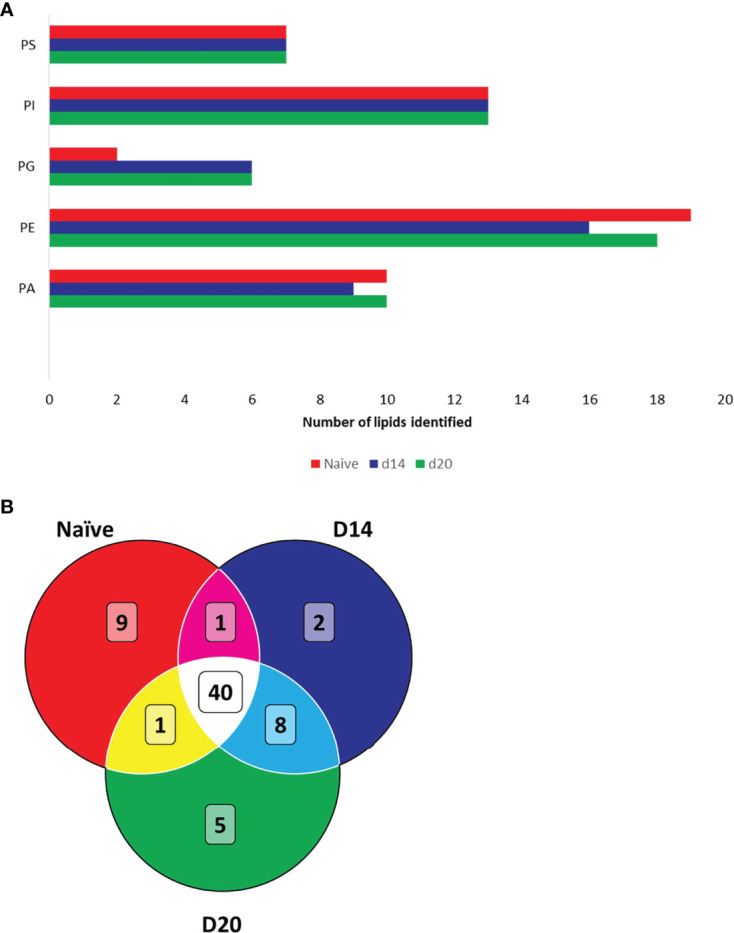
Structural identification of lipids using FTMS. **(A)** Lipids identified in naïve and infected mouse liver. Bar chart displays the total number of identified lipids per lipid class. **(B)** Venn diagram showing overlap of identified lipids in naïve and infected liver. Phosphatidic acids (PA), phosphatidylethanolamines (PE), phosphatidylinositols (PI), phosphatidylserines (PS) and phosphatidylglycerols (PG). Lipids are detailed in **Table S1**.

We next structurally identified the observed spatially-resolved lipid masses using tandem MS (see **Figure S5** for examples of tandem MS spectra). A total of 51, 51 and 54 spatially-distributed lipids from naïve, d14 and d20 mouse liver, respectively, were structurally identified (**Table S1**). A variety of glycerophospholipids were identified, subdivided into five different classes, namely, phosphatidic acids (PA), phosphatidylethanolamines (PE), phosphatidylinositols (PI), phosphatidylserines (PS), and phosphatidylglycerols (PG) (**Figure 4A**). Of these, PGs were mainly identified in the infected liver, whereas the other lipid classes exhibited differential numbers of identified lipids between naïve and infected livers. 40 lipids were common to all tissues, 8 were unique to infected tissue at both time points (2 PGs, 2 PIs and 2 PEs and 2 PAs), 5 unique to d20 (1 PA, 2 PGs 1 PE and 1 PI), 9 were unique to naïve tissue (4 PEs, 3 PAs, 1 PC and 1 PI) and one (PE) was common between naïve and d20 infected tissue (**Figure 4B**). Lipids are color-coded to the corresponding position in the venn diagram in **Figure 4B** in **Table S1**.

### Pathology-guided MSI characterizes heterogeneity of inflammatory microenvironments

**Figure 5:**
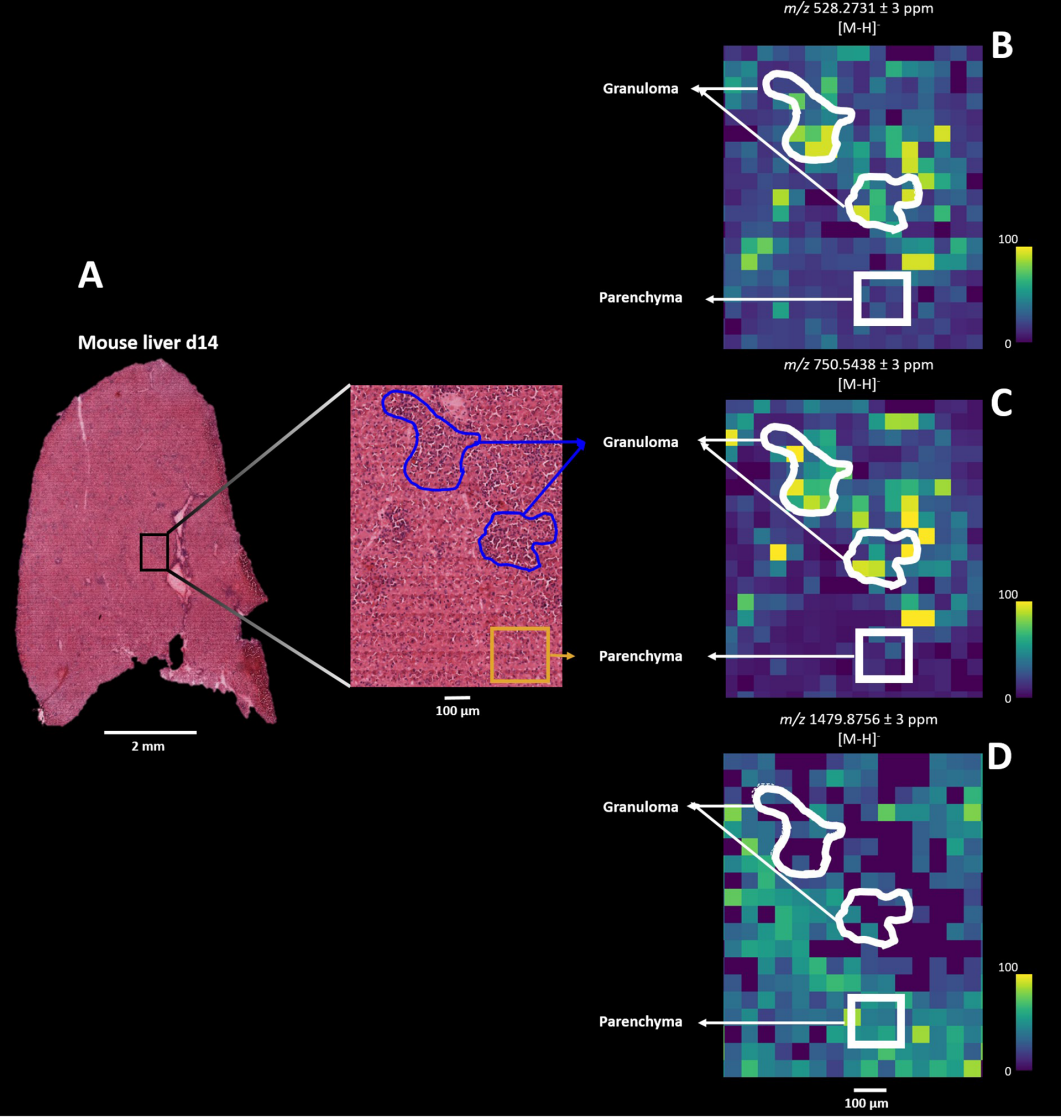
Pathology-guided mass spectrometry imaging workflow. Figure shows the ability to deduce analyzed lipid features from morphologic regions of interest (ROI): **(A)** Zoomed-in histopathological characterization of granulomas (blue) and surrounding parenchyma (yellow) in *L. donovani*-infected mice liver (d14 p.i.). H&E-stained images are co-registered with mass spectrometry images for subsequent spatial lipid analysis from annotated granuloma and parenchymal ROIs. **(B)** Spatial distribution of *m/z* 528.2731 ± 3 ppm, **(C)** *m/z* 750.5438 ± 3 ppm and **(D)** 1479.8756 ± 3 ppm from the granuloma and parenchymal ROIs.

Lipids are proposed to play a key role in host-parasite responses during *Leishmania* infection and both reflect and regulate local immunometabolism (10, 14, 51). Since we structurally identified spatially- resolved lipids, we next sought to delineate the dynamics of lipid compositional change in *L. donovani*-induced granulomas comparing directly between d14 and d20 p.i. In contrast to the data-driven results described above, we here deployed a pathology-guided MSI workflow in which all mouse liver samples were H&E stained for subsequent histopathological examination. This targeted approached allowed us to deduce which specific lipids were spatially distributed across granulomas and moreover identify potential lipid candidates underlying *L. donovani* pathogenesis. Regions of granulomatous inflammation were manually annotated, providing 10 granuloma and 10 parenchymal regions of interest (ROI; each ∼ 150 x 150 µm) for all d14 and d20 infected mouse livers (n=5 mice and 100 ROIs per time point; **Figure 5A**) for further comparison. Subsequent co-registration of H&E images with their respective MS images allowed us to deduce the measured lipid masses from the annotated ROIs (**Figure 5A**). Only those pixels that fell within the rim of the manually drawn granulomatous or parenchymal ROI were used for further analysis. As an example, we observed that *m/z* 528.2731 ± 3 ppm and *m/z* 750.5438 ± 3 ppm showed higher pixel intensities for the granuloma ROIs than parenchymal ROIs in a d14 parasitized mouse liver, whereas for *m/z* 1479.8576 ± 3 ppm this pattern was reversed (**Figures 5B–D**). Subsequently, we observed the same pixel intensity patterns from granuloma and parenchymal ROIs for these three masses in all other d14 and d20 parasitized mouse livers (**Figure S6**). In addition, based on the lipid composition from the annotated ROIs, we observed a clear discrimination by PCA between all 10 parenchymal and 10 granuloma ROIs, as shown for d14 infected liver depicted in **Figure 5A** (**Figure S7**).

**Figure 6:**
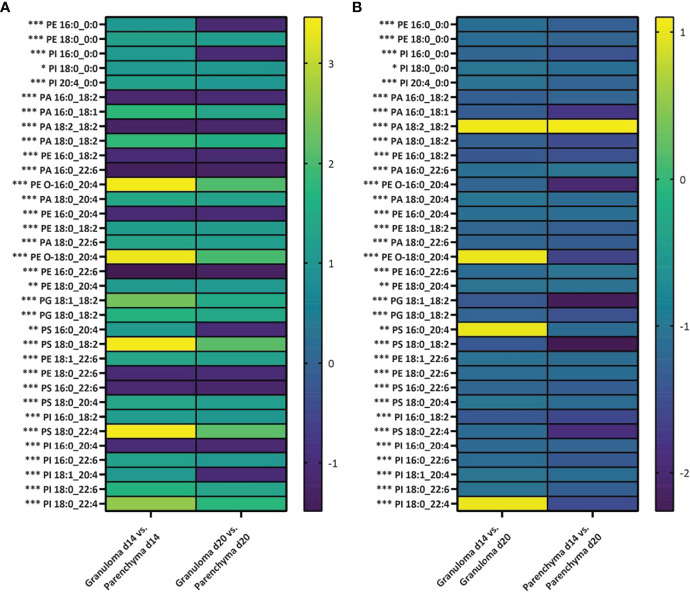
Differing abundance of lipids in granulomas and parenchyma over time of infection. Log2 fold changes in average relative intensities of lipids deduced from granulomas and parenchyma regions of interest (ROIs) from d14 and d20 p.i. (n=5 mice and 100 granulomatous and parenchymal ROIs per time point). **(A)** For each lipid, the fold change is presented for lipid intensity in granuloma ROIs compared to parenchymal ROIs for d14 and d20 parasitized mouse livers. **(B)** For each lipid, the fold change is presented for lipid intensity from granuloma or parenchymal d14 ROIs compared to granuloma or parenchymal d20 ROIs. Significance is defined as *p<0.05, **p<0.01 or ***p<0.001. P-values are adjusted for multiple comparisons. PA, phosphatidic acids; PE, phosphatidylethanolamines; PC, phosphocholine; PI, phosphatidylinositols; PS, phosphatidylserines; and PG, phosphatidylglycerols.

Next, we set out to evaluate how distinctive the lipid features were in these different microenvironments (*i.e.*, granuloma vs. parenchyma). All lipid intensities were extracted from all granuloma and parenchymal ROIs (n=5 mice and 100 ROIs at each time point) for subsequent ANOVA fold-change analysis. In general, intensities from PA, PE, PI, PS and PG lipid classes were either significantly increased or decreased in granuloma ROIs compared to parenchymal ROIs (**Figure 6A**). Interestingly, PE 16:0_0:0, PI 16:0_0:0, PS 16:0_20:4 and PI 18:1_20:4 showed a significantly increased intensity in a d14 granuloma ROIs compared to its surrounding parenchymal ROIs, whereas the intensities for these lipids were lower in d20 granuloma ROIs compared to parenchymal ROIs (**Figure 6A** and **Table S2**). Furthermore, we evaluated how the lipid intensities changed between d14 to d20 granuloma ROIs and parenchymal ROIs. Apart from PA 18:2_18:2, PE O-18:0_20:4, PS 16:0_20:4 and PI 18:0_22:4, all lipids showed a significant lower intensity in d14 granuloma ROIs versus d20 granuloma ROIs. In addition, all lipids, except for PA 18:2_18:2 showed a significant lower intensity in d14 parenchymal ROIs versus d20 parenchymal ROIs (**Figure 6B** and **Table S3**). These findings demonstrated dynamic changes of specific lipid intensities, linked to the evolution of granulomatous inflammation and that were apparent in both granulomas per se and within the surrounding parenchyma. Interestingly, 26% (9/34) of the identified lipids contained arachidonic acid (identified as the 20:4 chain at the *sn*-2 position), which is an important precursor for downstream inflammatory cascades induced by arachidonic acid derivatives (52).

## Discussion

Host-pathogen interactions are heterogeneous and result in dynamic changes of immunometabolic responses in local inflammatory microenvironments within the same infected tissue (53). Here, we have evaluated the spatial distributions of lipids in an experimental *L. donovani* mouse model to deduce the dynamics of local lipid compositions at two different time points of infection. *Leishmania* parasites are known to alter host lipid metabolism (10, 11, 54), however studies that have examined local tissue responses during visceral leishmaniasis are scarce. Our study demonstrates aberrant lipid compositions and intensities between granulomas and the surrounding parenchyma as well as dynamic lipid intensity changes in both granulomas and parenchyma at different time points of infection.

MSI is a powerful tool to study spatial lipid compositions underlying morphological characteristics related to *L. donovani* infection. Granuloma formation is a clinical hallmark of *L. donovani* infection (and leishmaniasis in general) (42), with patchy distributions across the affected tissue. Earlier research demonstrated the ability of MSI to visualize candidate peptides derived from visceralising *Leishmania* in mice (55) and to relate peptides to cutaneous *Leishmania* infection in mice (56). A recent study by Kloehn et al. (41) showed unique lipids distributed across the lesion induced by *L. mexicana* and surrounding host tissue. However, to our knowledge, there is no evidence that highlights lipid compositional changes between inflammatory microenvironments at different time points of infection. Our experimental model allowed us to evaluate the dynamics of spatially-resolved lipids in infected mice at different times post infection. The concomitant comparison to the spatial distribution of lipids from a naïve mouse liver highlighted several lipid masses that had either increased or decreased intensities in the d14 and d20 infected liver although subsequent analysis revealed that relatively few were unique to naive (9 lipids) or infected (15 lipids) tissue. Moreover, these lipids showed aberrant intensities in a similar patchy pattern to that of the distribution of granulomas.

To associate whether specific lipids were spatially resolved in relation to morphological characteristics of the tissue, *i.e.*, granulomas, high-resolution MALDI-MSI and subsequent H&E staining was deployed on a d14 parasitized mouse liver. We were able to correlate unique lipids to granulomatous and parenchymal regions, which suggests a tight microenvironmental control over lipid abundance likely related to cellular composition. Subsequently, we structurally identified lipids using tandem MS identifying specific lipid classes at each time point studied. We here focused on ionizing lipids in negative mode, as this allows detection of lipid classes that are highly related to more generalizable host-pathogen inflammatory interactions (14, 41, 57).

Several reviews highlight how leishmaniasis affects and causes aberrant lipid compositions (10, 11, 54). In addition, the role of lipids in these cascades are prominent as phospholipids (PLs), glycolipids (GLs) and cholesterol were shown to influence host-pathogen interactions following infection with *L. donovani* (41, 58). Interestingly, while most lipids exhibited either an increased or decreased intensity across infected compared to naïve liver, we also identified lipids that were mainly expressed in d14 and d20 parasitized livers (*viz.* PI 16:0_0:0, PG 16:1_16:1, PE O-16:0_22:6, PG 18:1_18:2, PE O-18:0_22:4, PG 20:4_18:0 and PI 18:0_18:1). Earlier research demonstrated that both promastigotes and amastigotes of *L. donovani* produce PC, PE, PI, PS, PG and PA lipid classes in various quantities (14). Furthermore, spatial distributions of PI 20:4 and PI 18:0_22:6 were attributed to either granulomatous regions or surrounding tissue, respectively in a study by Kloehn et al. (41). Analysis of adjacent tissue sections demonstrated that 45-71% of granulomas in the mouse livers analyzed likely contained amastigotes, but a limitation of our current methodology is that parasite quantitation was not directly evaluated in the specific granulomas assessed by MSI. Future protocol improvements may overcome this limitation and allow correlation between changes in lipid content and parasite abundance within individual granulomas.

Of note, in the infected mouse liver we observed a three-fold increase in the number of lipids identified belonging to the PG class, with PG 16:1_18:1, PG 18:1_18:2, PG 18:2_20:4, PG 16:0_18:2, PG 18:0_20:4 and PG 20:4_18:0 being unique to infected tissue (**Figure 4B** and **Table S1**). These results might hint towards potential candidate lipid biomarkers that are underlying of the host-pathogen interaction. Further research, however, is needed to identify both the upstream pathways responsible for changes in lipid composition (*e.g.* host transcriptomic changes associated with altered lipid biosynthesis) and their downstream consequences (*e.g.* in terms of host protection vs parasite survival). Such studies may also indicate whether changes in specific lipids may have prognostic potential. PGs are converted by a series of enzymatic conversions, *viz.* from PA to cytidine diphosphate diacylglycerol (CDP-DAG) by CDP-DAG synthase, to phosphatidylglycerophosphate (PGP) by PGP synthase and finally into PG by phosphatase (59). While we cannot deduce the origin of the increased number of PG lipids, *i.e.*, whether these PG lipids derive from the *Leishmania* parasite or not, there is evidence where host PG synthesis increases upon inflammatory responses. For example, obesity-induced gut microbiota and adipose tissue inflammation are associated with marked changes of PG production, linking its role to host metabolism and inflammation (60). In other examples, the role of PGs have been linked to modulating innate immunity and viral infections. Experimental evidence has suggested a suppressive role for PGs in respiratory infections like respiratory syncytial virus infection and the subsequent inflammatory host response (61). Furthermore, during *Mycoplasma pneumonia* infection PG suppresses pathogen-induced arachidonic acid release and thereby its subsequent downstream conversion into pro-inflammatory oxylipins (62). In addition, administration of palmitoyl-oleoyl PG (POPG), which is the most abundant surfactant phospholipid (63), *via* the airways of mice drastically alleviates Influenza A virus-induced inflammation (64). In contrast to the identification of six unique PGs in infected compared to naïve tissue, when we applied pathology-guided MSI only PG 18:1_18:2 was detected as more abundant in granulomas compared to the parenchyma, along with an additional PG, PG 18:0_18:2. This suggests that in addition to changes in PG abundance in granulomas, other changes to PG abundance may occur in the parenchyma. Further work will be required to identify the localization of these PGs and more generally on the role of PGs in modulating the *Leishmania* host-pathogen interaction. It is important to note that our analysis does not cover the full liver lipidome and further research expanding the breadth of analysis to other lipid classes is required in order to understand the full complexity of the *Leishmania*-pathogen interaction.

Our lipid datasets also uncover a potential link to relevant lipid-induced inflammatory cascades, such as downstream arachidonic acid (AA) metabolism to oxylipins, which are potent pro-inflammatory mediators (52, 65). AA is a n-6 polyunsaturated fatty acid (FA) and can be built onto the *sn-2* position of PE, PC and PI or is present as free AA (FA 20:4). Enzymatic conversion of AA by phospholipase A_2_ (PLA_2_) at the *sn-2* position gives rise to the 2-series cyclooxygenase (COX) oxylipins, *viz.* into the major prostaglandins PGD_2_, PGE_2_, PGI_2_ and PGF_2α_ (52, 65–68). Prostaglandins are involved in host-pathogen interactions (69) where PGE_2_ was shown to play a prominent role in the host-pathogen interaction in *Francisella novicida* (57) and *L. donovani* infections (70). We observed higher intensities of several PE, PI, PA, PS and PG lipid classes containing AA, identified as the 20:4 chain, in granulomas compared to surrounding parenchyma as well as higher intensities in d20 versus d14 granulomas compared to surrounding parenchyma. These results corroborate that AA-containing phospholipids are spatially resolved in *L. donovani*-induced inflammatory microenvironments and may serve as important precursors for downstream COX-2 oxylipins. With respect to the important role of AA metabolism, we did not identify AA nor any of the downstream converted metabolites. Free AA’s main function is to serve as a substrate for COX-2 oxylipins due to its cytotoxicity (65). The rapid processing of free AA into COX-2 oxylipins might explain its absence in our lipid dataset. Furthermore, the low abundance and instability of oxylipins make them challenging to analyze as they are prone to ion suppression during MALDI-MSI analysis (71). Of note, our previous transcriptomic analysis of the hepatic response to *L. donovani* (72) indicated reduced abundance of mRNA for lysophosphatidylglycerol acyltransferase 1 (*Lpgat1*) and increased abundance of mRNA for phospholipase A2 group IID (*Pla2g2d*) and phospholipase A2 group IVA (*Pla2g4a*). These results are in line with our observations of increased number of PG lipids in the infected liver, since LPGAT1 catalyzes the conversion of lysophosphatidylglycerol to phosphatidylglycerol (73). Furthermore, the increased expression of the phospholipase genes highlights the downstream conversion of AA into COX-2 oxylipins, since both PLA2G2D and PLA2G4A favor hydrolysis of the ester bond of the fatty acyl group at the *sn-2* position. Moreover, PLA2G2D specifically favors hydrolyzation of PE and PG (74, 75). A complementary spatial transcriptomic analysis to our current dataset and samples would further elaborate on the mechanistic pathways which specific phospholipids gets hydrolyzed to generate AA and its further conversion into inflammatory lipid derivatives.

Of all the granulomatous infections, pulmonary tuberculosis has been most well studied from the perspective of alterations in lipid composition. However, unlike most examples of granulomatous inflammation, including that induced by *Leishmania* infection, tuberculosis lung granulomas are often associated with a necrosis-dependent stimulation of triglyceride-rich foamy macrophages (76, 77). Importantly, inhibition of triglyceride synthesis by pharmacological blockade of diacylglycerol-O-acyltransferase (DGAT1) lead to reduced bacterial burden in a mouse model (78), exemplifying the potential of lipid-targeted host directed therapy. Thus, whilst *Leishmania* granulomas may have distinct characteristics based on the nature of the pathogen and the ensuing host response, the identification of pathways regulating lipid compositional changes may likewise help identify new approaches for therapeutic intervention (79).

In summary, we here show distinctive spatial-temporal changes of lipid intensities and compositions within hepatic granulomas and the surrounding parenchyma in the livers of mice infected with *L. donovani*. Specifically, we identified aberrant AA-containing phospholipid distributions and intensities between granulomas and parenchyma at different time point of infection. Using MSI, this explorative study elaborates and visualizes the involvement of lipids in the evolving host response to *L. donovani* infection.

## Data availability statement

The datasets presented in this study can be found in online repositories. The names of the repository/repositories and accession number(s) can be found below: https://metaspace2020.eu/project/tans-2021, tans-2021.

## Ethics statement

The animal study was reviewed and approved by the University of York Animal Welfare and Ethics Review Board and performed under UK Home Office license (‘Immunity and Immunopathology of Leishmaniasis’ Ref # P49487014).

## Author contributions

RT performed the MALDI-MSI experiments, subsequent data-analysis and designed the manuscript. SD and ND conducted the animal experiments and helped with data interpretation and provided feedback to the manuscript. PP helped with MALDI-MSI data analysis and JC generated additional experimental data. GC and PO’T provided insights and feedback. PK and RH reviewed the manuscript and are project leaders and corresponding authors. All authors contributed to the article and approved the submitted version.

## Funding

This work was funded by the York-Maastricht Partnership program (https://www.maastrichtuniversity.nl/york-maastricht-partnership) and supported by a Wellcome Trust Senior Investigator Award to PK (WT104726). This research was also part of the M4I research program and received financial support from the Dutch Province of Limburg under the LINK program.

## Conflict of interest

The authors declare that the research was conducted in the absence of any commercial or financial relationships that could be construed as a potential conflict of interest.

## Publisher’s note

All claims expressed in this article are solely those of the authors and do not necessarily represent those of their affiliated organizations, or those of the publisher, the editors and the reviewers. Any product that may be evaluated in this article, or claim that may be made by its manufacturer, is not guaranteed or endorsed by the publisher.
