# Supplementary material for: Mass spectrometry imaging identifies altered hepatic lipid signatures during experimental Leishmania donovani infection
Source: Front Immunol. 2022 Jul 28;13:862104. doi: 10.3389/fimmu.2022.862104 (PMC9394181; doi:10.3389/fimmu.2022.862104)
Supplement: Supplementary file 1 [file DataSheet_1.docx]

**Supplement**

In addition to the naïve liver used as comparator against infected mouse livers, we assessed the consistency of lipid profiles and spatial distributions across five naïve mouse livers in total (figure S1). Related to the comparator (naïve liver old, facing upside down), the four additional mice showed similar lipid spectra across the 760-890 *m/z* range (figure S1A), where most lipids showed spatial distribution across the total tissue area. In addition, spatial distribution across all naïve livers showed homogenous abundances. The slightly observed variation of some lipid intensities might be explained by biological variation between individual mouse livers (figure S1B-F).

**
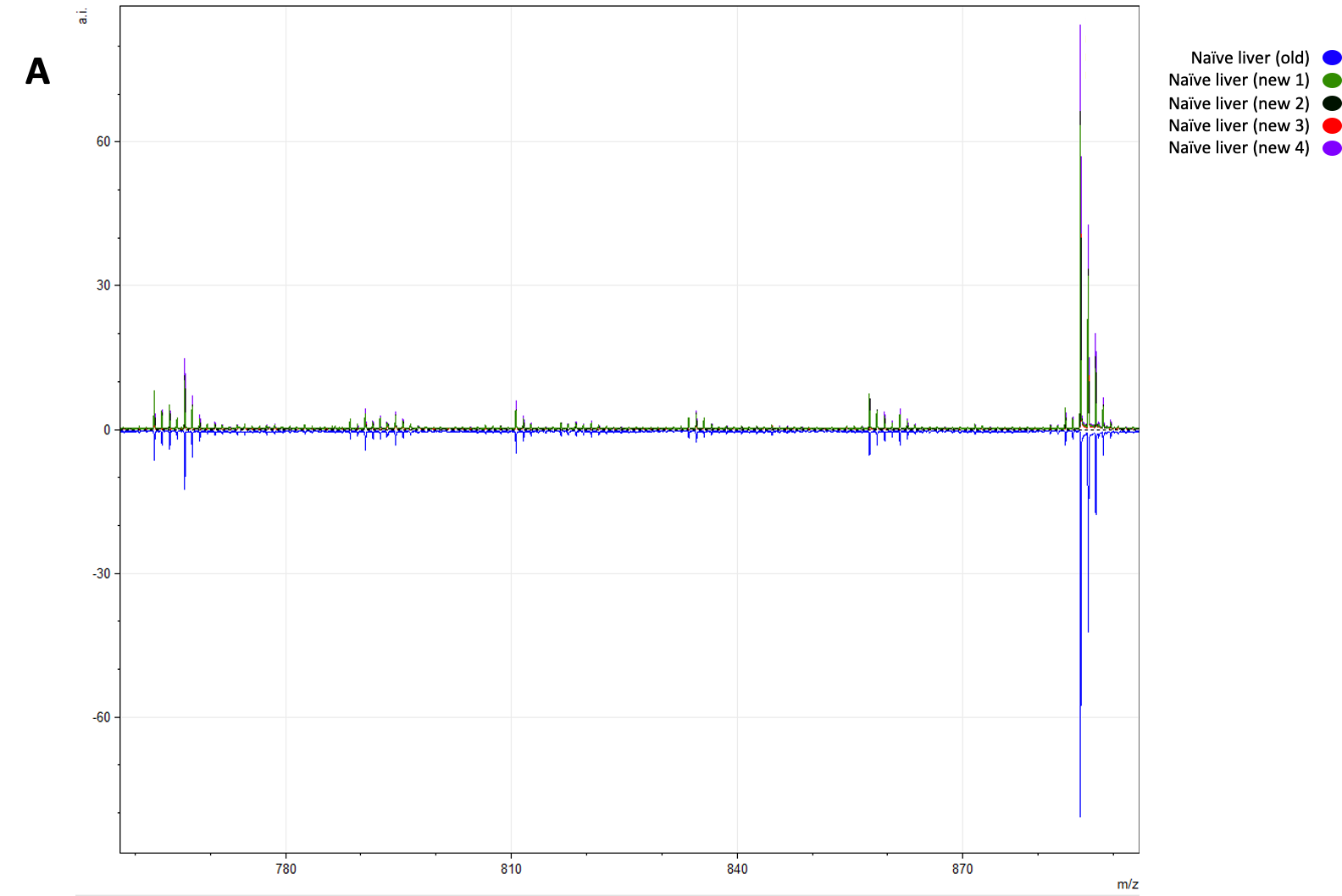
**

**
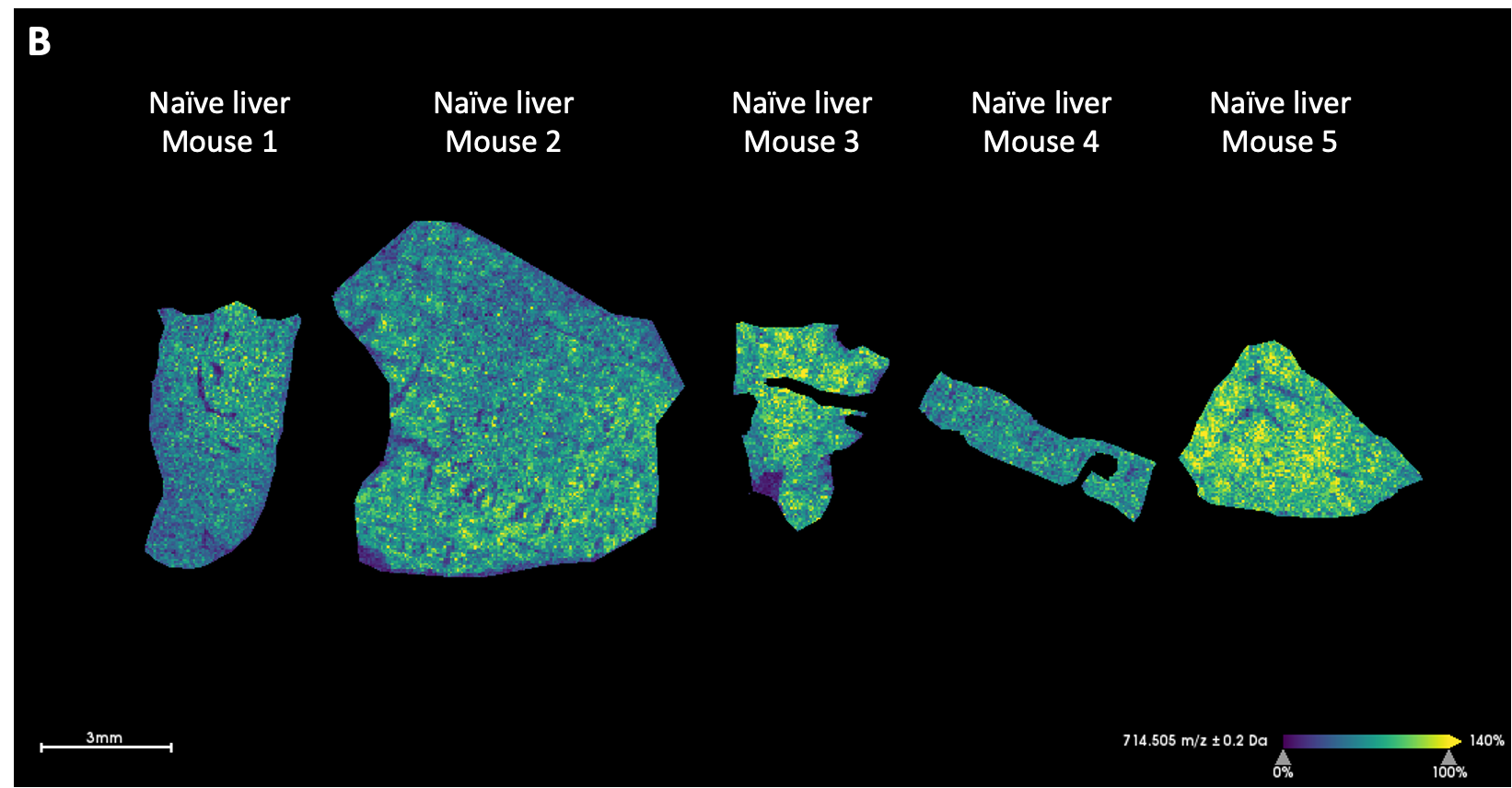
**

**
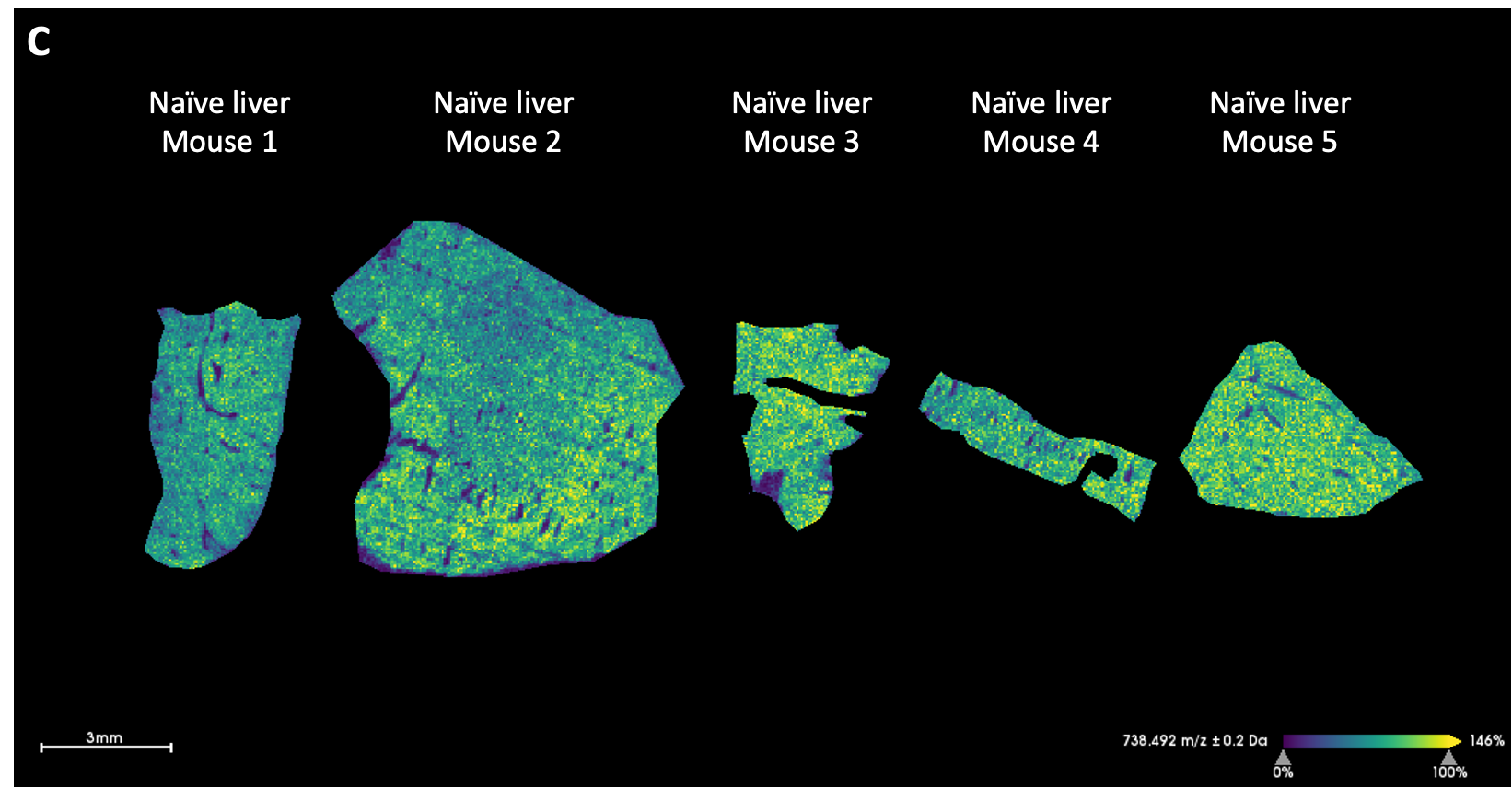
**

**
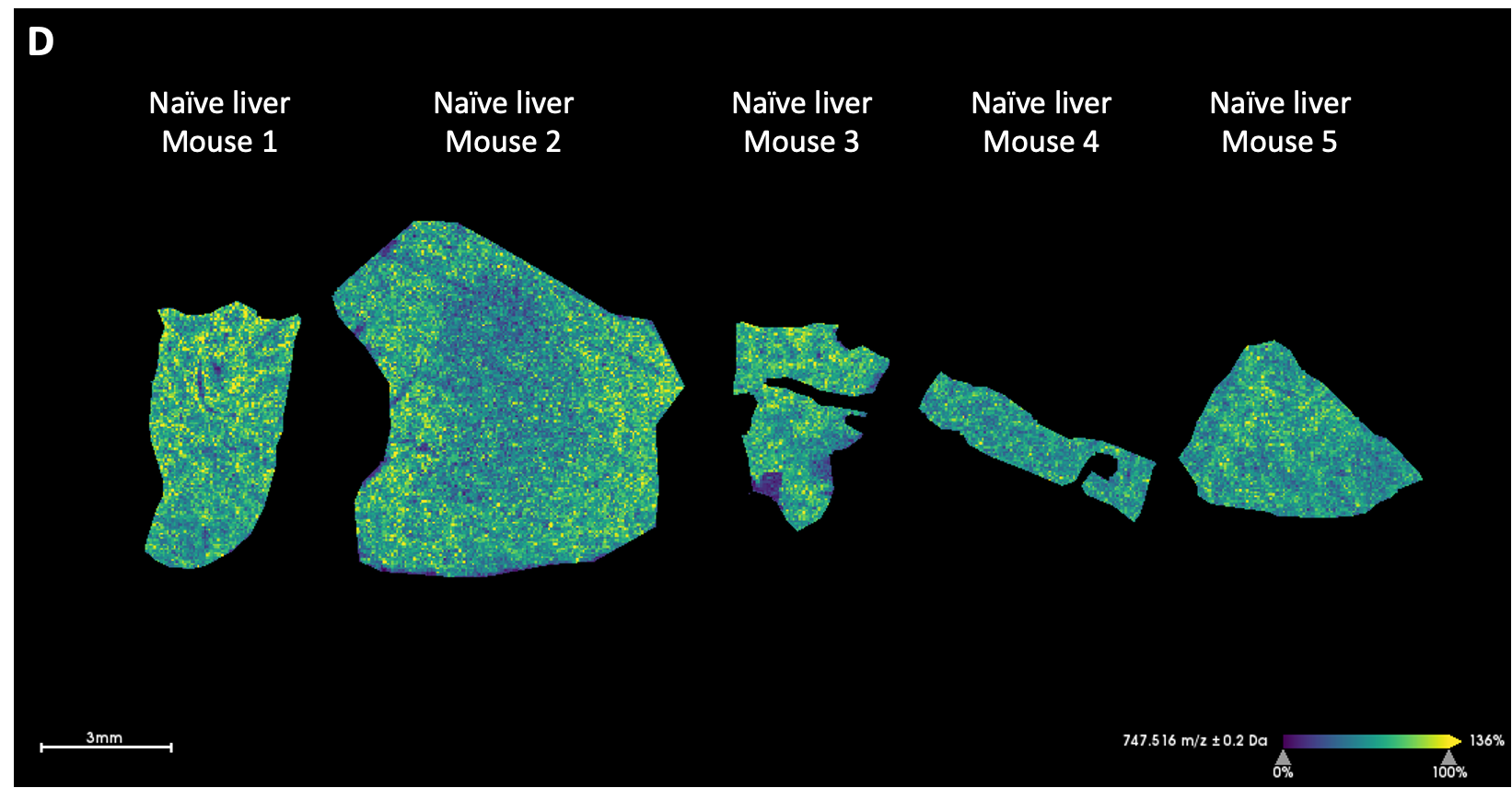
**

**
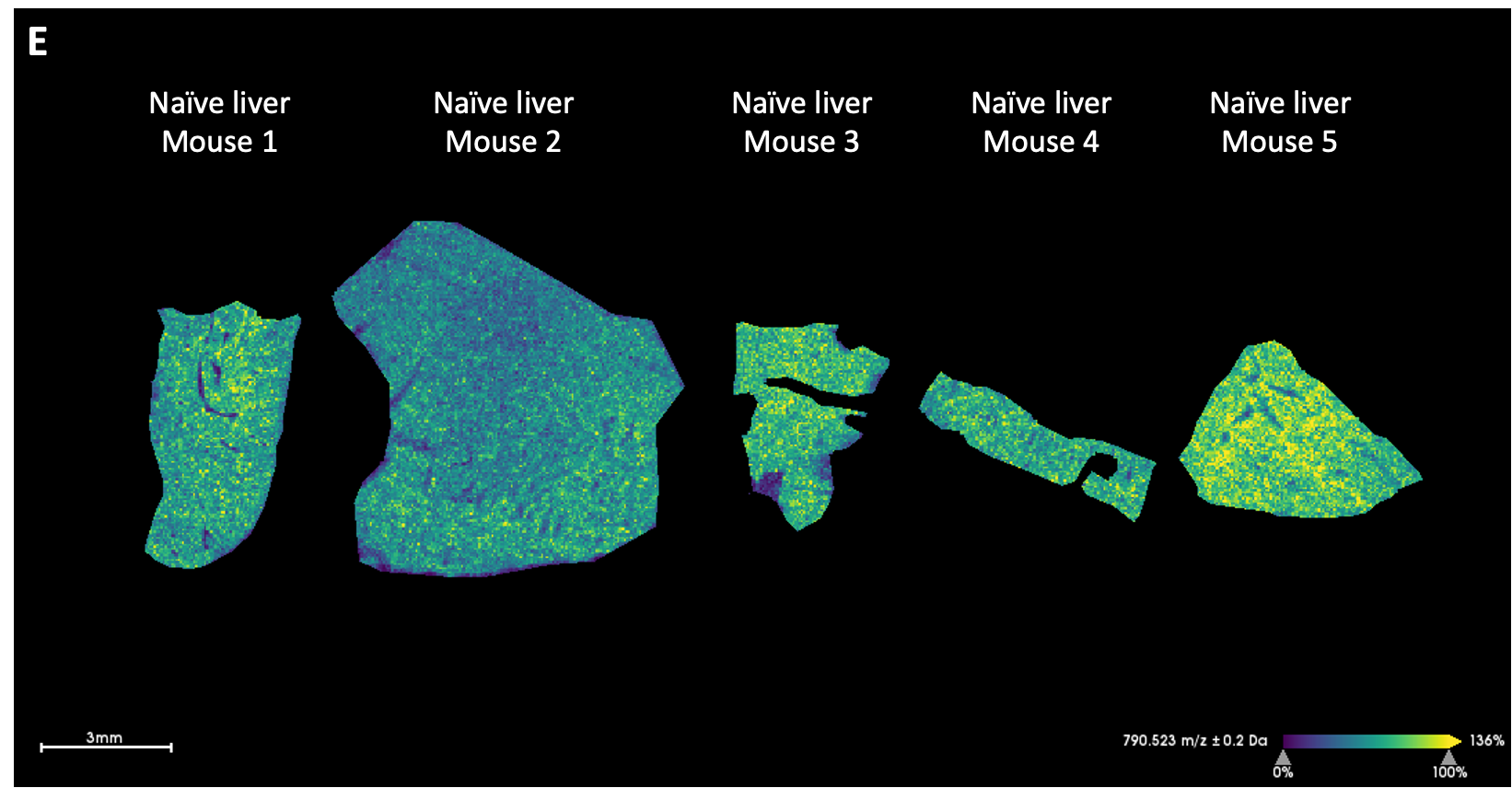
**

**
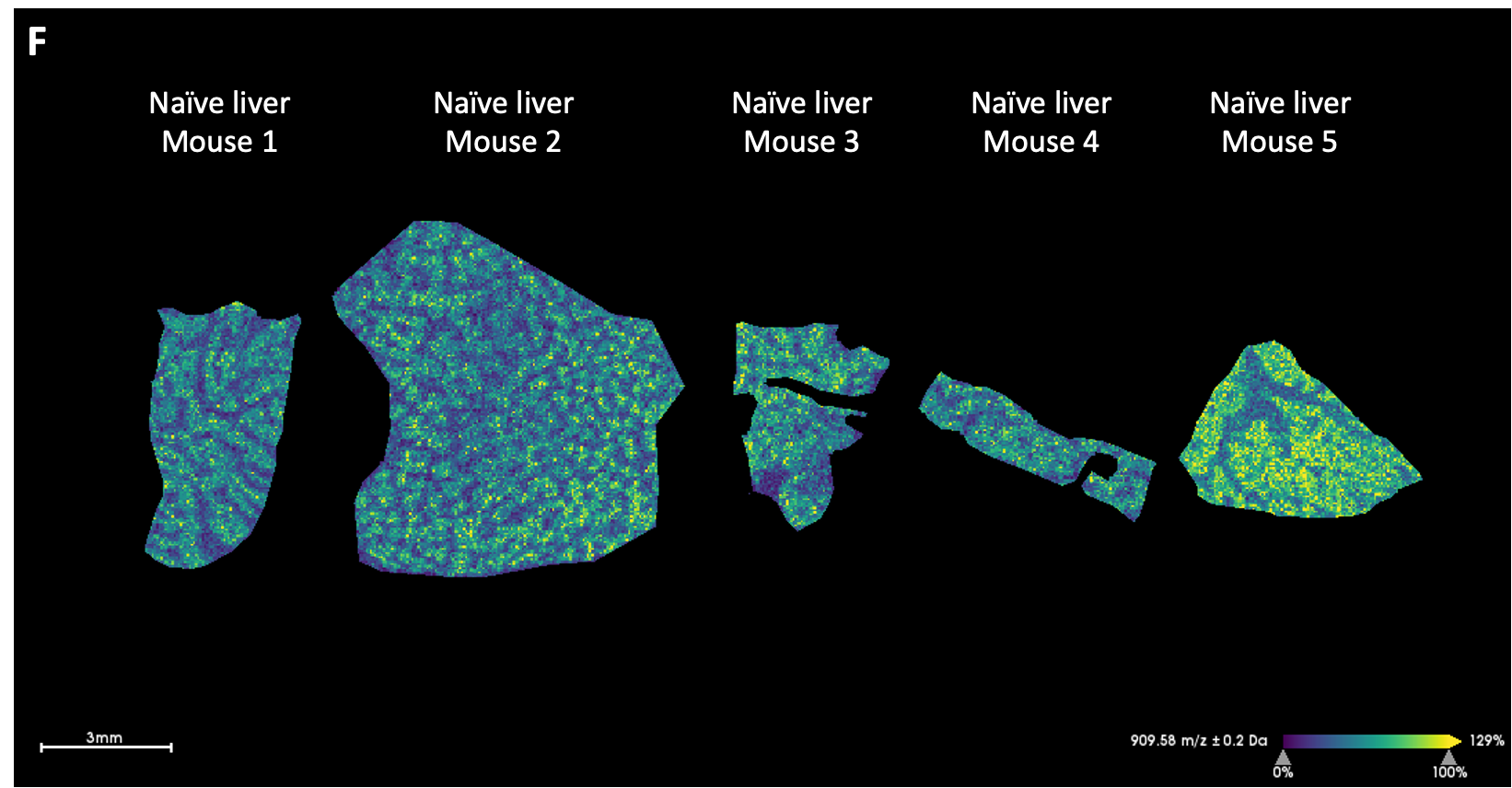
**

**Figure S1**: Naïve mouse livers (n=5) were obtained from (individual) naïve C57BL/6 mice. The old naïve liver (the comparator against the infected mouse livers) is compared to four additional (individual) naïve mouse livers. **A:** Mass spectra of 5 naïve mouse livers are shown in the 760 – 890 *m/z* range, where spectra of naïve. **B-F:** Spatial distributions of the relative abundances of *m/z* 714.5, 738.5, 747.5, 790.5 and 909.6 (± 0.2 Da), respectively, in five naïve livers are shown.


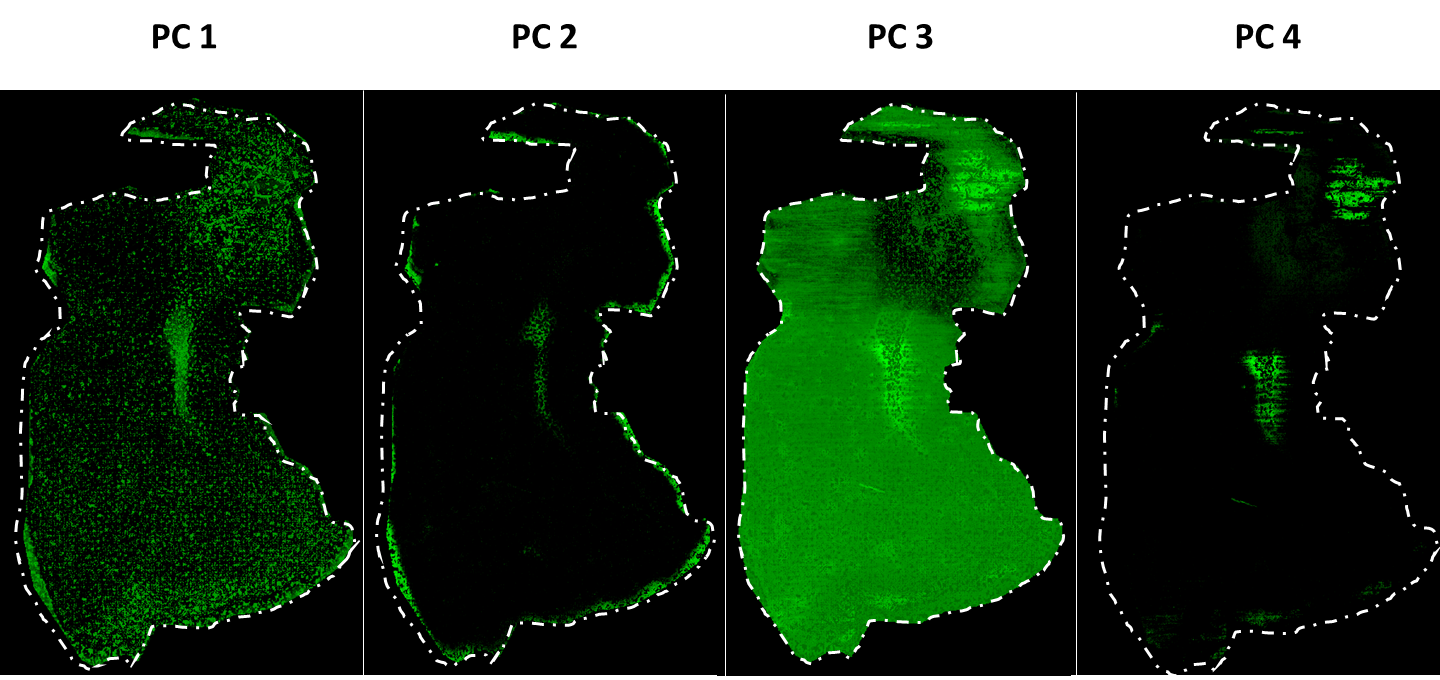
**Figure S2**: Principal component analysis (PCA) score image, of the first four principal components, shows the variance of spatially resolved lipid masses from a *Leishmanina donovani* - 14 days post-infected (d14) mouse liver that are not correlated to the morphological characteristics of the tissue.


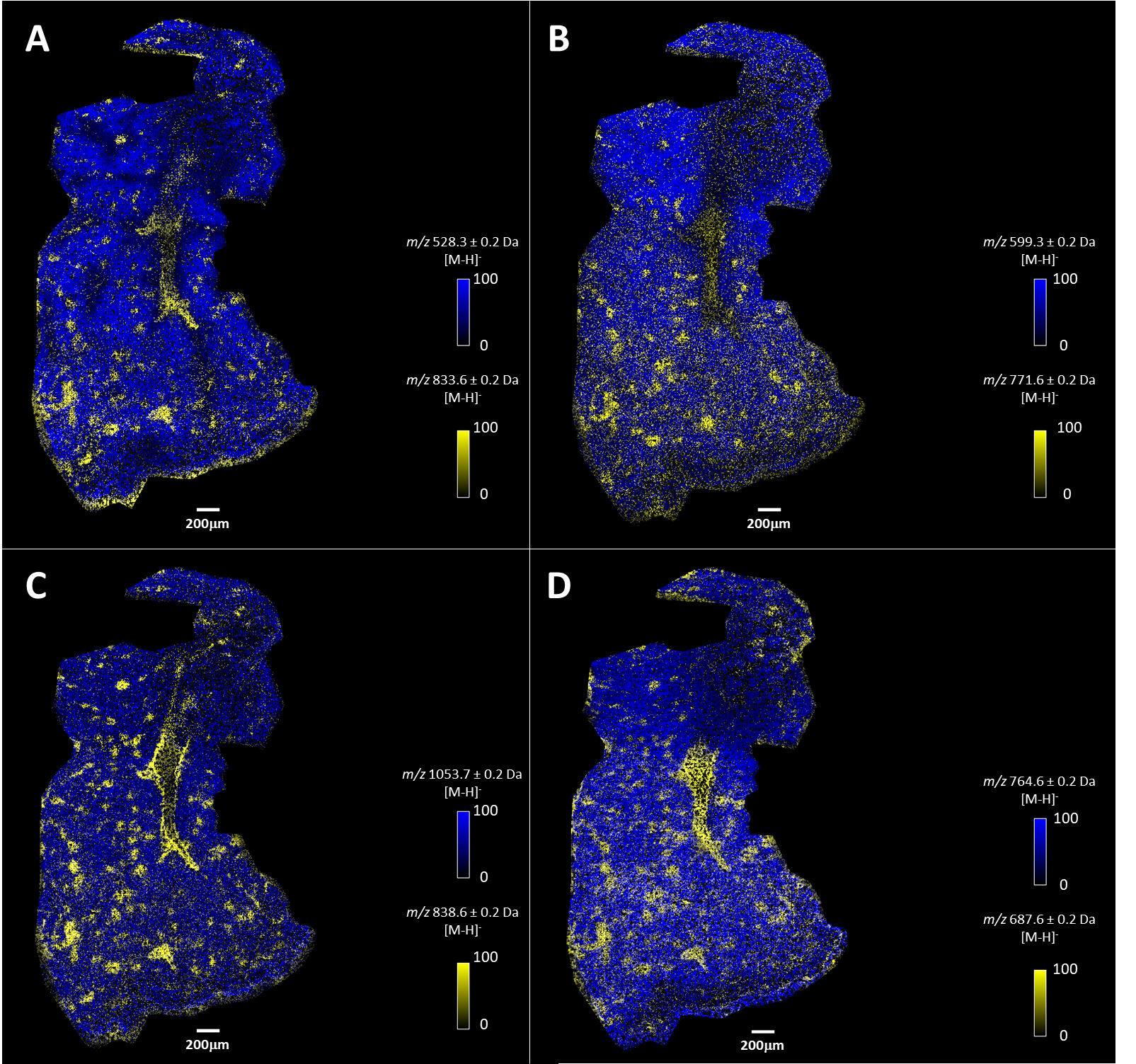
**Figure S3**: Overlaid images of the spatial distribution of the relative abundance of **(A)** *m/z* 528.3 and *m/z* 833.6; **(B)** *m/z* 599.3 and *m/z* 771.6; **(C)** *m/z* 1053.7 and *m/z* 838.6; **(D)** *m/z* 746.6 and *m/z* 687.6.


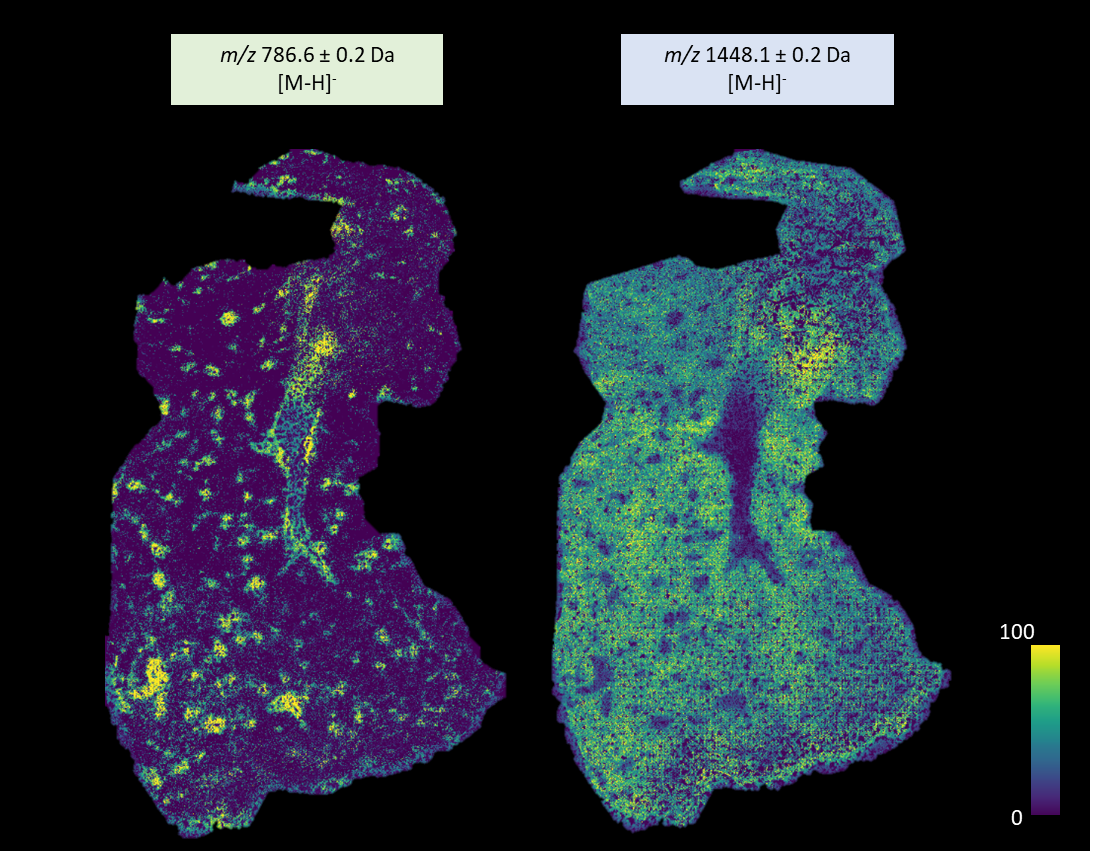


**Figure S4**: Spatial distribution of the relative abundance of *m/z* 786.6 and *m/z* 1448.1 ± 0.2 Da of a *Leishmanina donovani* - 14 days post-infected (d14) mouse liver.

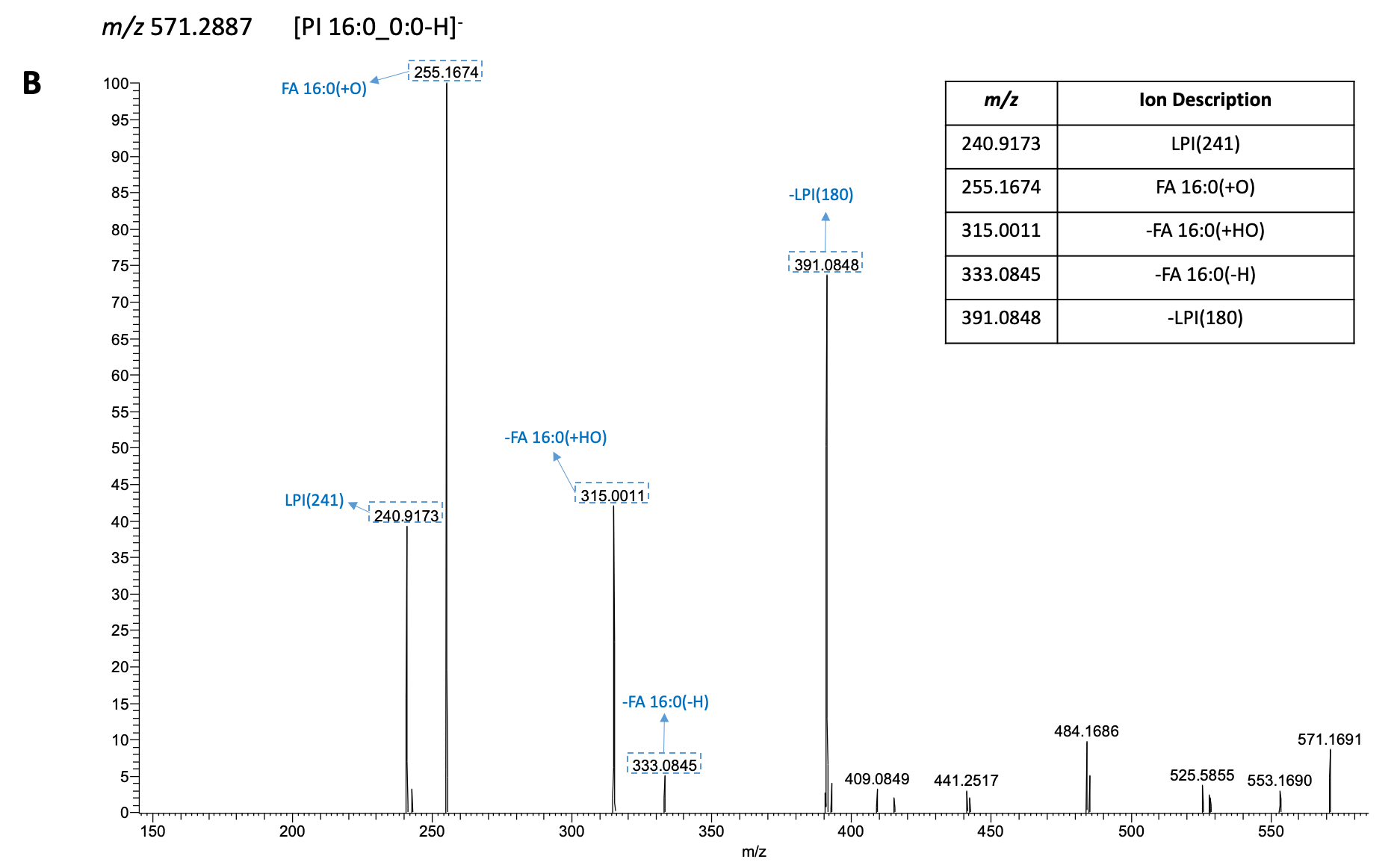

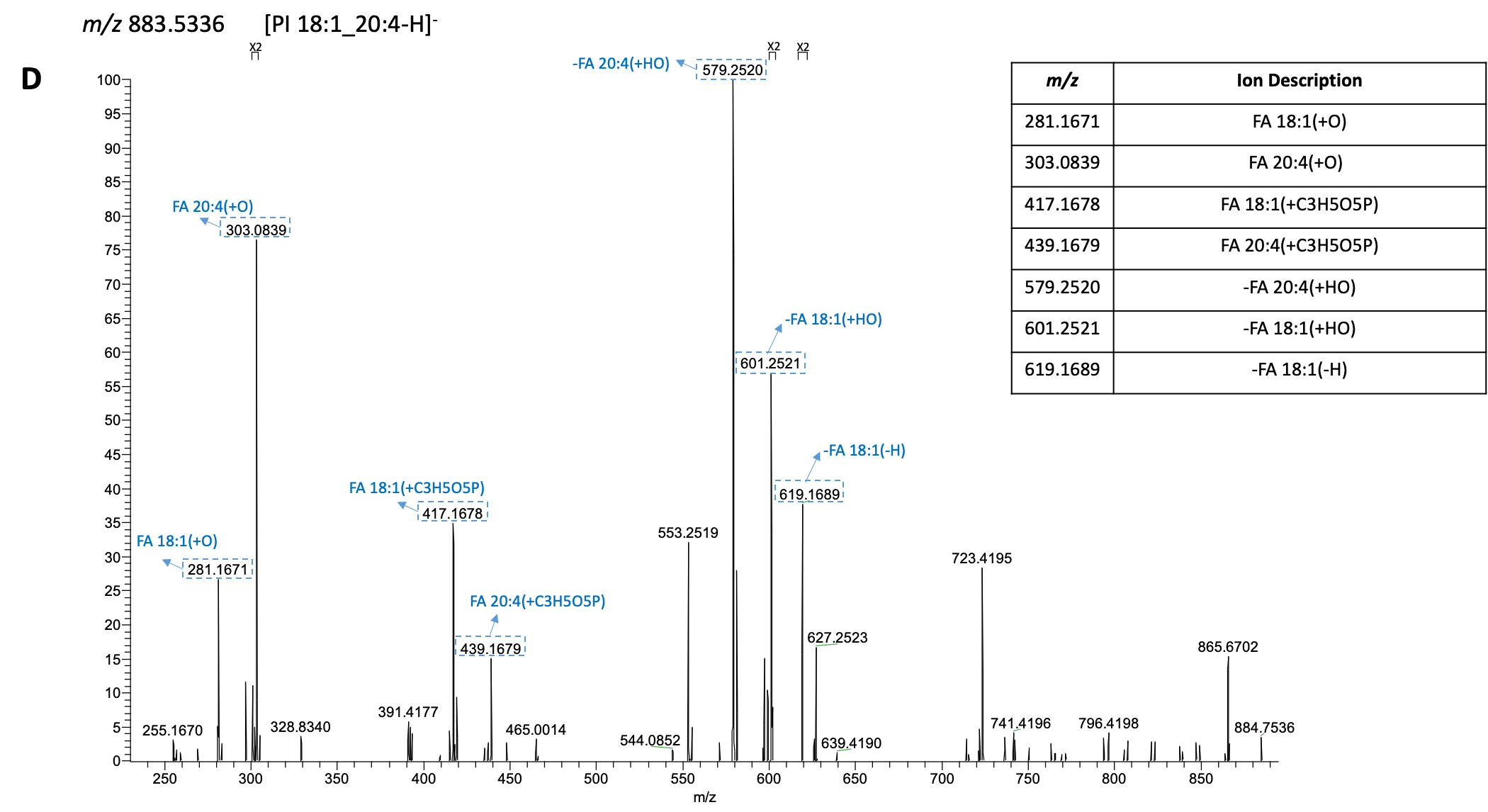


**Figure S5:** Tandem MS spectra for four different lipid species retrieved from IT-MS/MS data. **A – D** depict PE 16:0_0:0, PI 16:0_0:0, PS 16:0_20:4 and PI 18:1_20:4, respectively, with their respective ion description for each annotated *m/z* value.

**Table S1**: Accurate mass precursor lipid *m/z* including delta mass (ΔM) in parts per million (ppm) as well as its respective annotated lipid identifier (ID) and chemical formula. Lipid IDs are color-coded to the corresponding position in the venn diagram (figure 4E).

| **All identified lipid masses from naïve mouse liver** | | | | |
| --- | --- | --- | --- | --- |
| **Lipid precursor m/z** | **Adduct** | **ΔM ppm** | **Lipid ID** | **Chemical formula** |
| 437.2672 | [M-H]^-^ | 0.4 | PA 0:0_18:0 | C21H43O7P |
| 452.2782 | [M-H]^-^ | 0.2 | PE 16:0_0:0 | C21H44NO7P |
| 478.2939 | [M-H]^-^ | 0.1 | PE 18:1_0:0 | C23H46NO7P |
| 480.3093 | [M-H]^-^ | 0.6 | PE 18:0_0:0 | C23H48NO7P |
| 500.2782 | [M-H]^-^ | 0.1 | PE 20:4_0:0 | C25H44NO7P |
| 524.2788 | [M-H]^-^ | 1 | PE 22:6_0:0 | C27H44NO7P |
| 599.3197 | [M-H]^-^ | 0.9 | PI 18:0_0:0 | C27H53O12P |
| 619.2882 | [M-H]^-^ | 1.1 | PI 20:4_0:0 | C29H49O12P |
| 671.465 | [M-H]^-^ | 1.1 | PA 16:0_18:2 | C37H69O8P |
| 695.4649 | [M-H]^-^ | 1.2 | PA 16:0_20:4 | C39H69O8P |
| 697.4811 | [M-H]^-^ | 0.4 | PA 18:1_18:2 | C39H71O8P |
| 699.4962 | [M-H]^-^ | 1.2 | PA 18:0_18:2 | C39H73O8P |
| 714.507 | [M-H]^-^ | 1.4 | PE 16:0_18:2 | C39H74NO8P |
| 716.5224 | [M-H]^-^ | 1.7 | PE 16:0_18:1 | C39H76NO8P |
| 719.4649 | [M-H]^-^ | 1.2 | PA 16:0_22:6 | C41H69O8P |
| 721.4805 | [M-H]^-^ | 1.3 | PA 18:1_20:4 | C41H71O8P |
| 722.5126 | [M-H]^-^ | 0.5 | PE O-16:0_20:4 | C41H74NO7P |
| 723.4962 | [M-H]^-^ | 1.2 | PA 18:0_20:4 | C41H73O8P |
| 725.511 | [M-H]^-^ | 2.3 | PA 18:0_20:3 | C41H75O8P |
| 736.4919 | [M-H]^-^ | 0.6 | PE 16:1_20:4 | C41H72NO8P |
| 738.507 | [M-H]^-^ | 1.3 | PE 16:0_20:4 | C41H74NO8P |
| 742.5383 | [M-H]^-^ | 1.3 | PE 18:0_18:2 | C41H78NO8P |
| 747.4961 | [M-H]^-^ | 1.2 | PA 18:0_22:6 | C43H73O8P |
| 750.5438 | [M-H]^-^ | 0.6 | PE O-18:0_20:4 | C43H78NO7P |
| 762.507 | [M-H]^-^ | 1.3 | PE 16:0_22:6 | C43H74NO8P |
| 766.5381 | [M-H]^-^ | 1.4 | PE 18:0_20:4 | C43H78NO8P |
| 769.5039 | [M-H]^-^ | 1.7 | PG 18:2_18:2 | C42H75O10P |
| 770.5695 | [M-H]^-^ | 1.3 | PE 20:0_18:2 | C43H82NO8P |
| 773.5332 | [M-H]^-^ | 0.8 | PG 18:0_18:2 | C42H79O10P |
| 782.4967 | [M-H]^-^ | 1.4 | PS 16:0_20:4 | C42H74NO10P |
| 786.5285 | [M-H]^-^ | 0.8 | PS 18:0_18:2 | C42H78NO10P |
| 788.5224 | [M-H]^-^ | 1.5 | PE 18:1_22:6 | C45H76NO8P |
| 790.5381 | [M-H]^-^ | 1.4 | PE 18:0_22:6 | C45H78NO8P |
| 792.5313 | [M+Cl]^-^ | 0.3 | PC 18:0_16:2 | C42H80NO8P |
| 793.5226 | [M-H]^-^ | 1.3 | PI O-16:0_16:1 | C41H79O12P |
| 794.5694 | [M-H]^-^ | 1.4 | PE 20:0_20:4 | C45H82NO8P |
| 806.4966 | [M-H]^-^ | 1.5 | PS 16:0_22:6 | C44H74NO10P |
| 808.5136 | [M-H]^-^ | 0.2 | PS 18:1_20:4 | C44H76NO10P |
| 810.528 | [M-H]^-^ | 1.3 | PS 18:0_20:4 | C44H78NO10P |
| 818.5701 | [M-H]^-^ | 0.6 | PE 20:0_22:6 | C47H82NO8P |
| 833.5174 | [M-H]^-^ | 1.4 | PI 16:0_18:2 | C43H79O13P |
| 834.5275 | [M-H]^-^ | 1.8 | PS 18:0_22:6 | C46H78NO10P |
| 838.5598 | [M-H]^-^ | 0.7 | PS 18:0_22:4 | C46H82NO10P |
| 857.5174 | [M-H]^-^ | 1.3 | PI 16:0_20:4 | C45H79O13P |
| 861.5487 | [M-H]^-^ | 1.4 | PI 18:0_18:2 | C45H83O13P |
| 881.5181 | [M-H]^-^ | 0.5 | PI 16:0_22:6 | C47H79O13P |
| 883.5331 | [M-H]^-^ | 1.3 | PI 18:1_20:4 | C47H81O13P |
| 885.5485 | [M-H]^-^ | 1.5 | PI 18:0_20:4 | C47H83O13P |
| 909.5484 | [M-H]^-^ | 1.6 | PI 18:0_22:6 | C49H83O13P |
| 911.5648 | [M-H]^-^ | 0.8 | PI 18:0_22:5 | C49H85O13P |
| 913.5796 | [M-H]^-^ | 1.7 | PI 18:0_22:4 | C49H87O13P |
|  |  |  |  |  |
| **All identified lipid masses from inflamed mouse liver d14** | | | | |
| **Lipid precursor m/z** | **Adduct** | **ΔM ppm** | **Lipid ID** | **Chemical formula** |
| 437.2673 | [M-H]^-^ | 0.1 | PA 0:0_18:0 | C21H43O7P |
| 452.2782 | [M-H]^-^ | 0.1 | PE 16:0_0:0 | C21H44NO7P |
| 480.3095 | [M-H]^-^ | 0 | PE 18:0_0:0 | C23H48NO7P |
| 500.2783 | [M-H]^-^ | 0 | PE 20:4_0:0 | C25H44NO7P |
| 524.279 | [M-H]^-^ | 1.4 | PE 22:6_0:0 | C27H44NO7P |
| 571.2887 | [M-H]^-^ | 0.3 | PI 16:0_0:0 | C25H49O12P |
| 599.32 | [M-H]^-^ | 0.3 | PI 18:0_0:0 | C27H53O12P |
| 619.2886 | [M-H]^-^ | 0.5 | PI 20:4_0:0 | C29H49O12P |
| 671.4654 | [M-H]^-^ | 0.5 | PA 16:0_18:2 | C37H69O8P |
| 695.4653 | [M-H]^-^ | 0.6 | PA 18:2_18:2 | C39H69O8P |
| 697.481 | [M-H]^-^ | 0.5 | PA 18:0_18:3 | C39H71O8P |
| 699.4966 | [M-H]^-^ | 0.6 | PA 18:0_18:2 | C39H73O8P |
| 714.5075 | [M-H]^-^ | 0.6 | PE 16:0_18:2 | C39H74NO8P |
| 719.4653 | [M-H]^-^ | 0.6 | PA 16:0_22:6 | C41H69O8P |
| 721.4811 | [M-H]^-^ | 0.4 | PA 18:1_20:4 | C41H71O8P |
| 722.5125 | [M-H]^-^ | 0.8 | PE O-16:0_20:4 | C41H74NO7P |
| 723.4966 | [M-H]^-^ | 0.6 | PA 18:0_20:4 | C41H73O8P |
| 738.5075 | [M-H]^-^ | 0.6 | PE 16:0_20:4 | C41H74NO8P |
| 742.5388 | [M-H]^-^ | 0.6 | PE 18:0_18:2 | C41H78NO8P |
| 745.5021 | [M-H]^-^ | 0.6 | PG 16:1_18:1 | C40H75O10P |
| 746.5114 | [M-H]^-^ | 2.1 | PE O-16:0_22:6 | C43H74NO7P |
| 747.4966 | [M-H]^-^ | 0.6 | PA 18:0_22:6 | C43H73O8P |
| 750.5437 | [M-H]^-^ | 0.9 | PE O-18:0_20:4 | C43H78NO7P |
| 762.5074 | [M-H]^-^ | 0.7 | PE 16:0_22:6 | C43H74NO8P |
| 766.5388 | [M-H]^-^ | 0.5 | PE 18:0_20:4 | C43H78NO8P |
| 769.5024 | [M-H]^-^ | 0.2 | PG 18:2_18:2 | C42H75O10P |
| 770.5699 | [M-H]^-^ | 0.8 | PE 20:0_18:2 | C43H82NO8P |
| 771.5181 | [M-H]^-^ | 0.1 | PG 18:1_18:2 | C42H77O10P |
| 773.5331 | [M-H]^-^ | 0.9 | PG 18:0_18:2 | C42H79O10P |
| 778.5748 | [M-H]^-^ | 1.1 | PE O-18:0_22:4 | C45H82NO7P |
| 782.4972 | [M-H]^-^ | 0.7 | PS 16:0_20:4 | C42H74NO10P |
| 786.5285 | [M-H]^-^ | 0.7 | PS 18:0_18:2 | C42H78NO10P |
| 788.5231 | [M-H]^-^ | 0.6 | PE 18:1_22:6 | C45H76NO8P |
| 790.5386 | [M-H]^-^ | 0.8 | PE 18:0_22:6 | C45H78NO8P |
| 793.502 | [M-H]^-^ | 0.7 | PG 18:2_20:4 | C44H75O10P |
| 797.5337 | [M-H]^-^ | 0.1 | PG 20:4_18:0 | C44H79O10P |
| 806.4972 | [M-H]^-^ | 0.7 | PS 16:0_22:6 | C44H74NO10P |
| 808.5129 | [M-H]^-^ | 0.6 | PS 18:1_20:4 | C44H76NO10P |
| 810.5285 | [M-H]^-^ | 0.7 | PS 18:0_20:4 | C44H78NO10P |
| 833.518 | [M-H]^-^ | 0.7 | PI 16:0_18:2 | C43H79O13P |
| 834.5281 | [M-H]^-^ | 1.1 | PS 18:0_22:6 | C46H78NO10P |
| 838.5596 | [M-H]^-^ | 0.9 | PS 18:0_22:4 | C46H82NO10P |
| 857.518 | [M-H]^-^ | 0.6 | PI 16:0_20:4 | C45H79O13P |
| 861.5494 | [M-H]^-^ | 0.5 | PI 18:0_18:2 | C45H83O13P |
| 863.5647 | [M-H]^-^ | 0.9 | PI 18:0_18:1 | C45H85O13P |
| 881.5179 | [M-H]^-^ | 0.7 | PI 16:0_22:6 | C47H79O13P |
| 883.5336 | [M-H]^-^ | 0.7 | PI 18:1_20:4 | C47H81O13P |
| 885.5492 | [M-H]^-^ | 0.8 | PI 18:0_20:4 | C47H83O13P |
| 909.5491 | [M-H]^-^ | 0.8 | PI 18:0_22:6 | C49H83O13P |
| 911.5647 | [M-H]^-^ | 0.9 | PI 18:0_22:5 | C49H85O13P |
| 913.5803 | [M-H]^-^ | 0.9 | PI 18:0_22:4 | C49H87O13P |
|  |  |  |  |  |
| **All identified lipid masses from inflamed mouse liver d20** | | | | |
| **Lipid precursor m/z** | **Adduct** | **ΔM ppm** | **Lipid ID** | **Chemical formula** |
| 437.2673 | [M-H]^-^ | 0.1 | PA 0:0_18:0 | C21H43O7P |
| 452.2782 | [M-H]^-^ | 0.1 | PE 16:0_0:0 | C21H44NO7P |
| 480.3094 | [M-H]^-^ | 0.3 | PE 18:0_0:0 | C23H48NO7P |
| 500.2781 | [M-H]^-^ | 0.4 | PE 20:4_0:0 | C25H44NO7P |
| 524.2781 | [M-H]^-^ | 0.2 | PE 22:6_0:0 | C27H44NO7P |
| 528.309 | [M-H]^-^ | 1.1 | PE 22:4_0:0 | C27H48NO7P |
| 571.2887 | [M-H]^-^ | 0.3 | PI 16:0_0:0 | C25H49O12P |
| 599.3199 | [M-H]^-^ | 0.5 | PI 18:0_0:0 | C27H53O12P |
| 619.2885 | [M-H]^-^ | 0.6 | PI 20:4_0:0 | C29H49O12P |
| 671.4656 | [M-H]^-^ | 0.2 | PA 16:0_18:2 | C37H69O8P |
| 673.4812 | [M-H]^-^ | 0.3 | PA 16:0_18:1 | C37H71O8P |
| 695.4652 | [M-H]^-^ | 0.7 | PA 18:2_18:2 | C39H69O8P |
| 697.4808 | [M-H]^-^ | 0.9 | PA 18:0_18:3 | C39H71O8P |
| 699.4965 | [M-H]^-^ | 0.8 | PA 18:0_18:2 | C39H73O8P |
| 714.5073 | [M-H]^-^ | 0.9 | PE 16:0_18:2 | C39H74NO8P |
| 719.4651 | [M-H]^-^ | 0.8 | PA 16:0_22:6 | C41H69O8P |
| 721.4811 | [M-H]^-^ | 0.3 | PA 18:1_20:4 | C41H71O8P |
| 722.5123 | [M-H]^-^ | 1 | PE O-16:0_20:4 | C41H74NO7P |
| 723.4966 | [M-H]^-^ | 0.7 | PA 18:0_20:4 | C41H73O8P |
| 738.5074 | [M-H]^-^ | 0.7 | PE 16:0_20:4 | C41H74NO8P |
| 742.5387 | [M-H]^-^ | 0.7 | PE 18:0_18:2 | C41H78NO8P |
| 745.5022 | [M-H]^-^ | 0.4 | PG 16:0_18:2 | C40H75O10P |
| 746.512 | [M-H]^-^ | 1.4 | PE O-16:0_22:6 | C43H74NO7P |
| 747.4965 | [M-H]^-^ | 0.7 | PA 18:0_22:6 | C43H73O8P |
| 750.5435 | [M-H]^-^ | 1.1 | PE O-18:0_20:4 | C43H78NO7P |
| 762.5074 | [M-H]^-^ | 0.7 | PE 16:0_22:6 | C43H74NO8P |
| 766.5387 | [M-H]^-^ | 0.6 | PE 18:0_20:4 | C43H78NO8P |
| 769.5022 | [M-H]^-^ | 0.4 | PG 18:2_18:2 | C42H75O10P |
| 770.5698 | [M-H]^-^ | 0.9 | PE 20:0_18:2 | C43H82NO8P |
| 771.5176 | [M-H]^-^ | 0.7 | PG 18:1_18:2 | C42H77O10P |
| 773.5329 | [M-H]^-^ | 1.2 | PG 18:0_18:2 | C42H79O10P |
| 778.5752 | [M-H]^-^ | 0.6 | PE O-18:0_22:4 | C45H82NO7P |
| 782.497 | [M-H]^-^ | 1 | PS 16:0_20:4 | C42H74NO10P |
| 786.5284 | [M-H]^-^ | 0.8 | PS 18:0_18:2 | C42H78NO10P |
| 788.5228 | [M-H]^-^ | 1 | PE 18:1_22:6 | C45H76NO8P |
| 790.5387 | [M-H]^-^ | 0.7 | PE 18:0_22:6 | C45H78NO8P |
| 793.5017 | [M-H]^-^ | 1 | PG 18:2_20:4 | C44H75O10P |
| 794.5697 | [M-H]^-^ | 1 | PE 20:0_20:4 | C45H82NO8P |
| 797.5331 | [M-H]^-^ | 0.8 | PG 18:0_20:4 | C44H79O10P |
| 806.497 | [M-H]^-^ | 1 | PS 16:0_22:6 | C44H74NO10P |
| 808.5128 | [M-H]^-^ | 0.7 | PS 18:1_20:4 | C44H76NO10P |
| 810.5285 | [M-H]^-^ | 0.7 | PS 18:0_20:4 | C44H78NO10P |
| 833.5179 | [M-H]^-^ | 0.7 | PI 16:0_18:2 | C43H79O13P |
| 834.5281 | [M-H]^-^ | 1.1 | PS 18:0_22:6 | C46H78NO10P |
| 838.5594 | [M-H]^-^ | 1.1 | PS 18:0_22:4 | C46H82NO10P |
| 857.5179 | [M-H]^-^ | 0.7 | PI 16:0_20:4 | C45H79O13P |
| 861.5492 | [M-H]^-^ | 0.8 | PI 18:0_18:2 | C45H83O13P |
| 863.5649 | [M-H]^-^ | 0.6 | PI 18:0_18:1 | C45H85O13P |
| 881.5177 | [M-H]^-^ | 1 | PI 18:2_20:4 | C47H79O13P |
| 883.5336 | [M-H]^-^ | 0.7 | PI 18:1_20:4 | C47H81O13P |
| 885.5492 | [M-H]^-^ | 0.8 | PI 18:0_20:4 | C47H83O13P |
| 909.5489 | [M-H]^-^ | 1.1 | PI 18:0_22:6 | C49H83O13P |
| 911.5645 | [M-H]^-^ | 1 | PI 18:0_22:5 | C49H85O13P |
| 913.5802 | [M-H]^-^ | 1.1 | PI 18:0_22:4 | C49H87O13P |


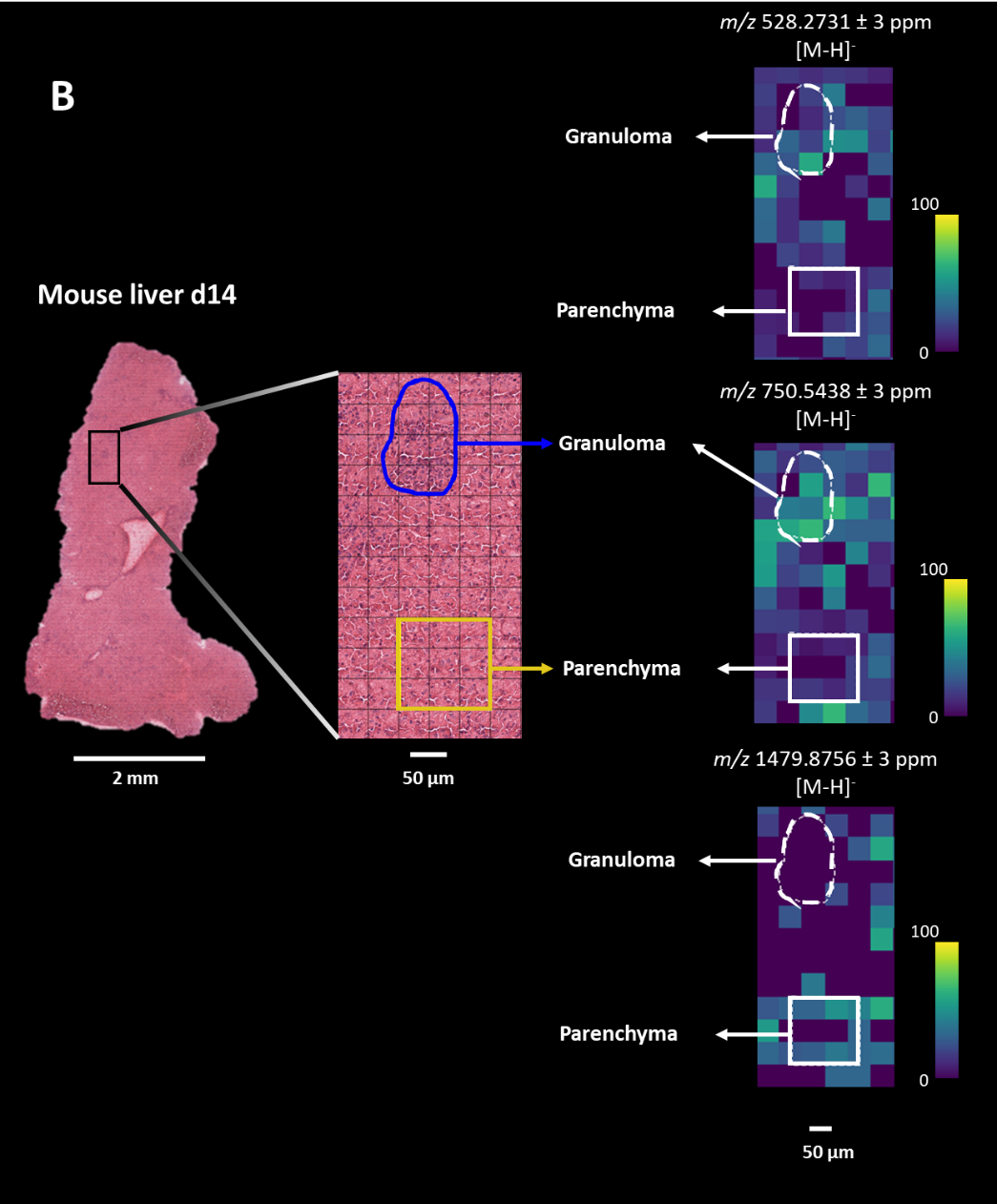

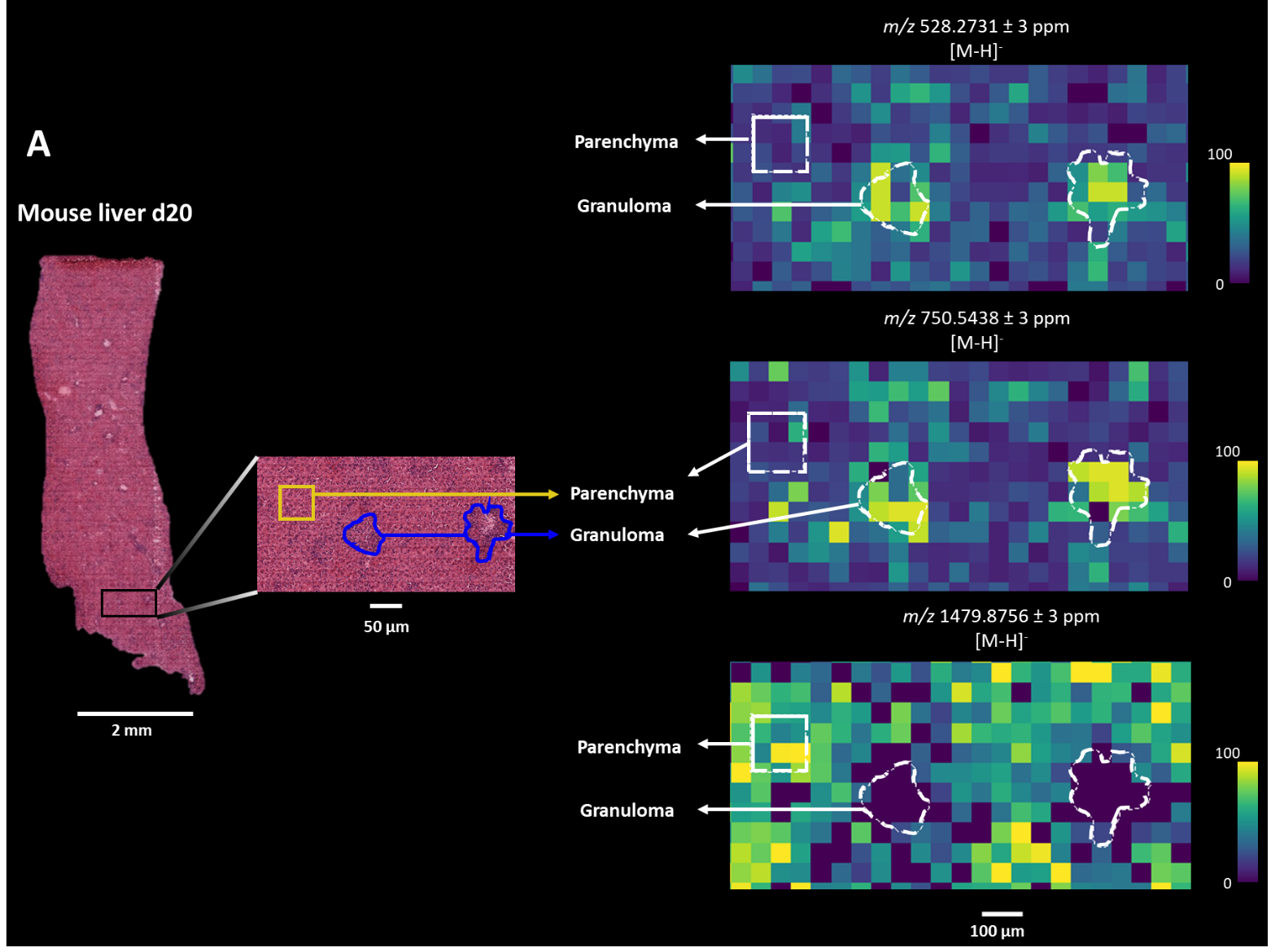


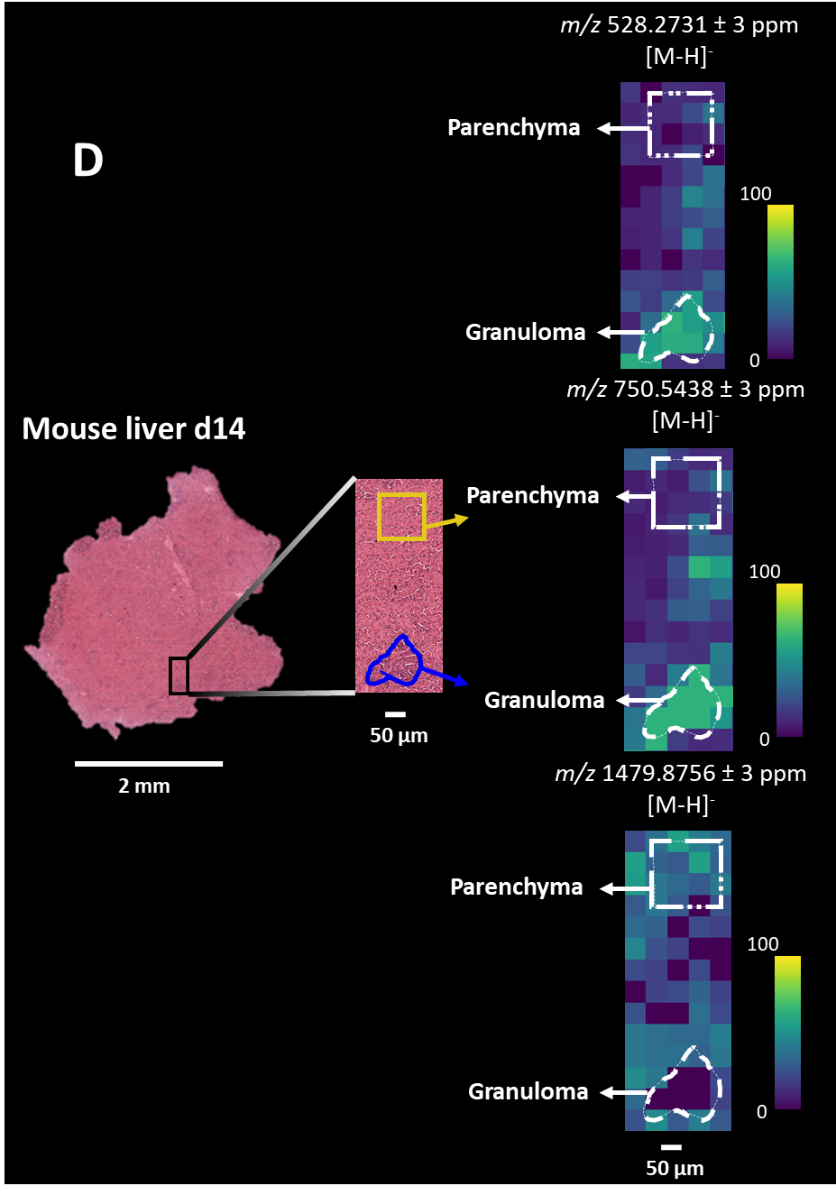

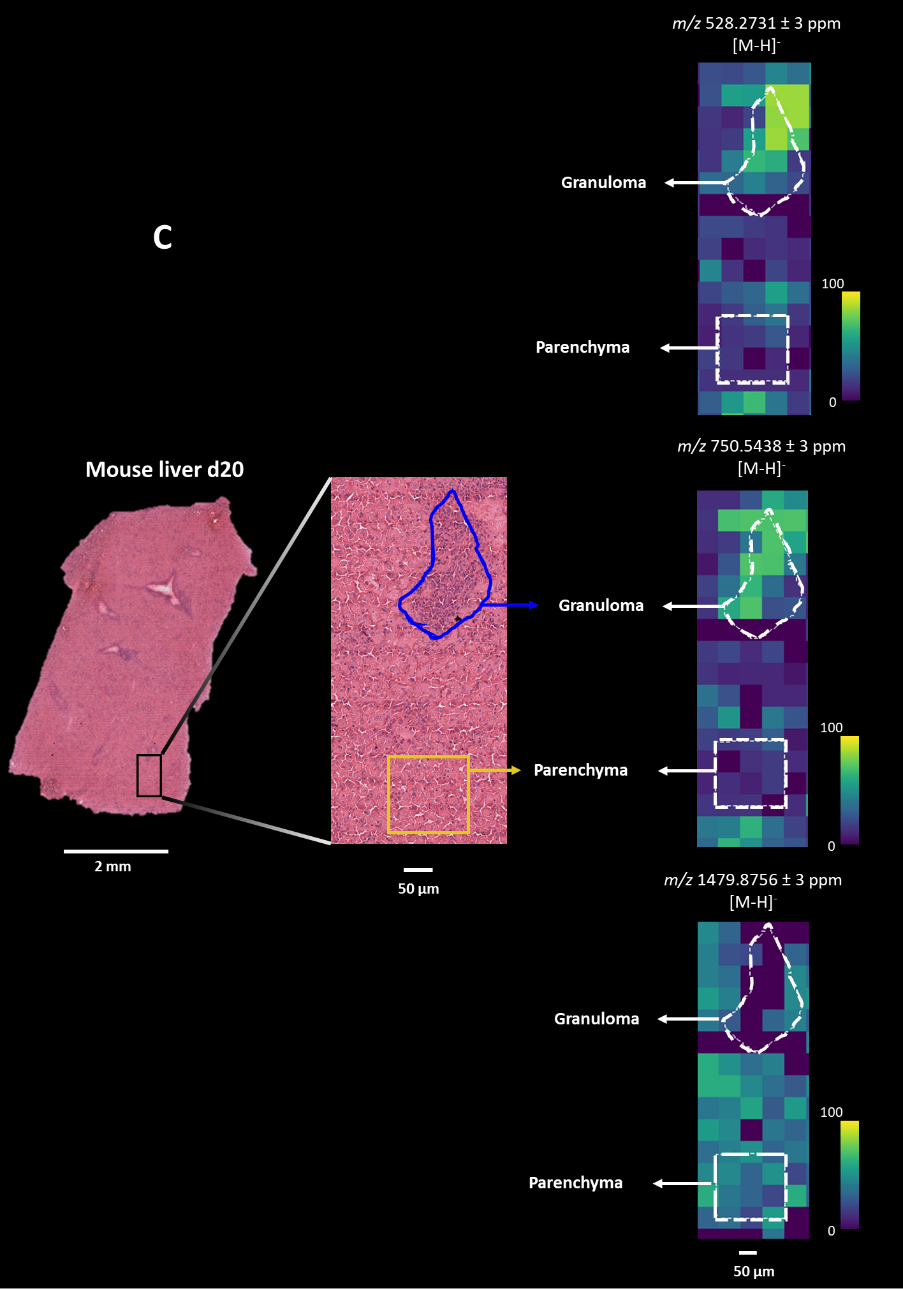


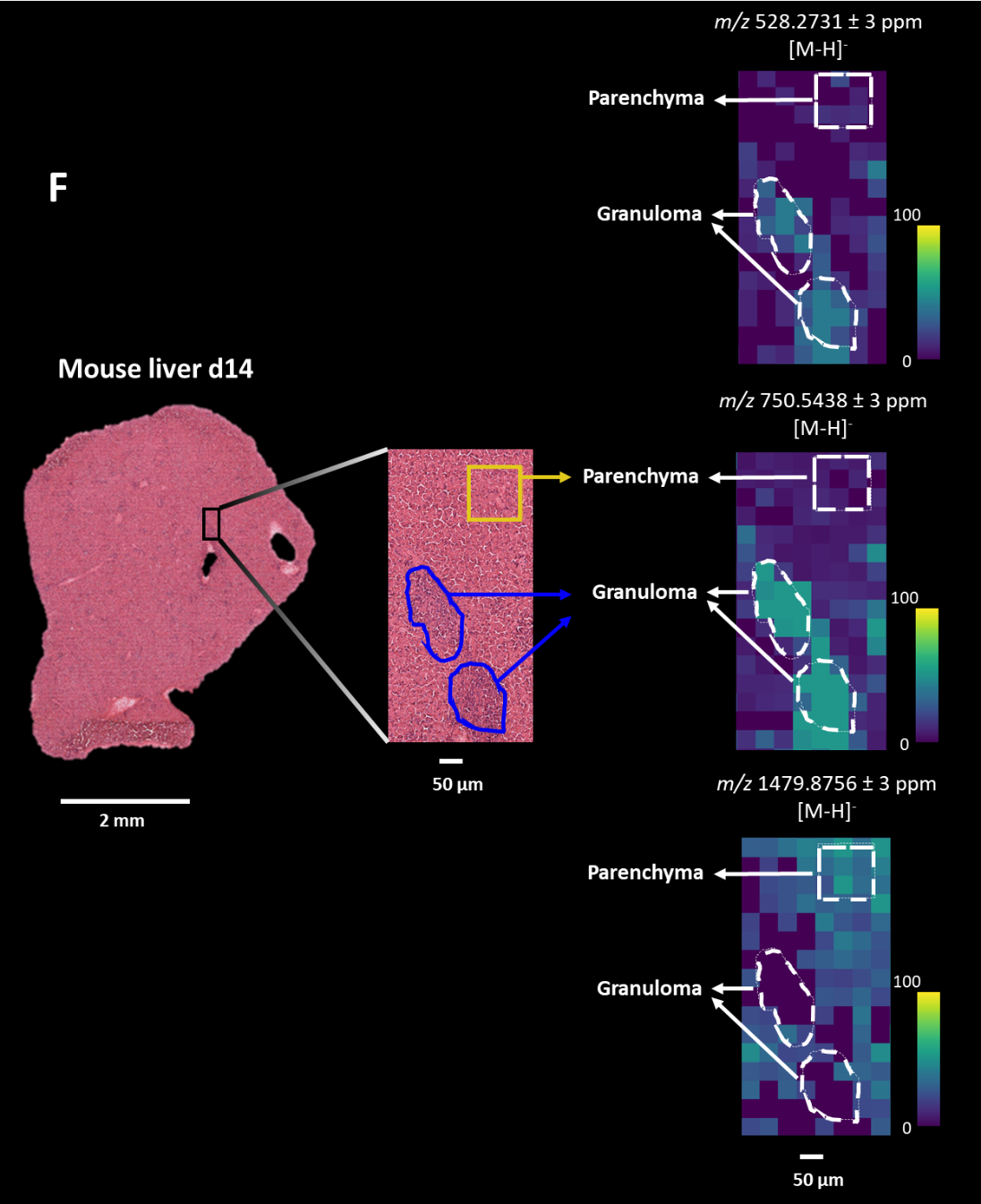

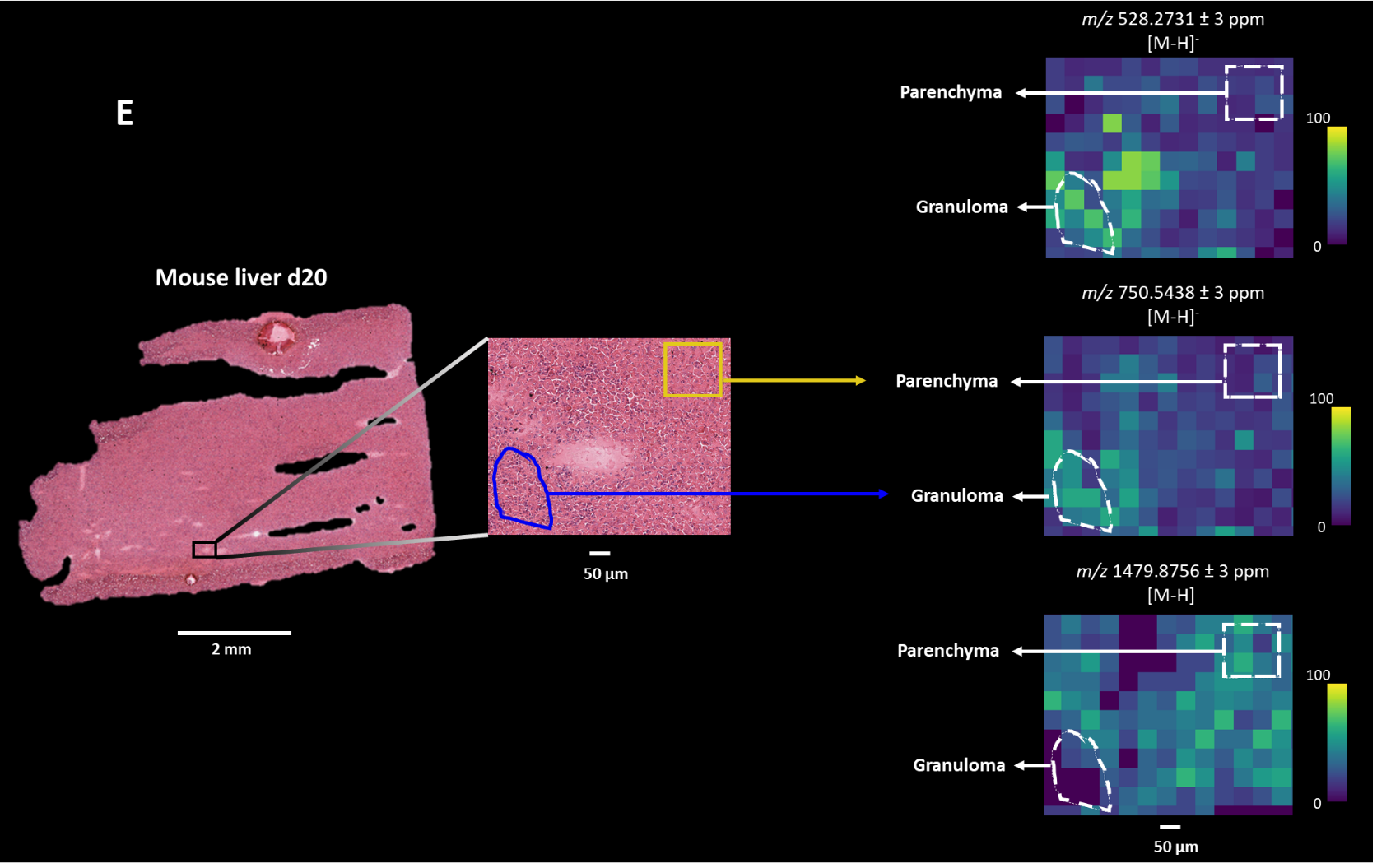


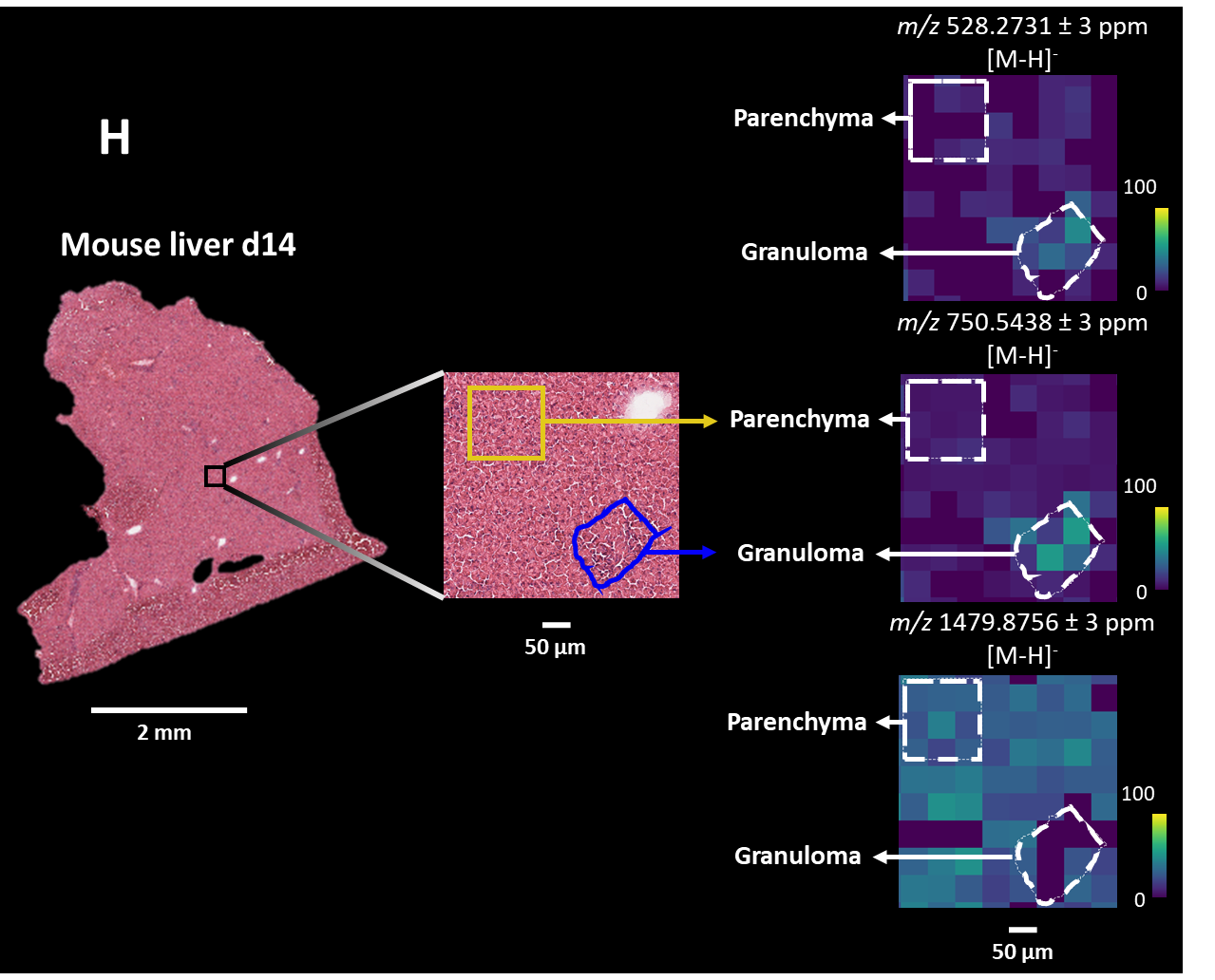

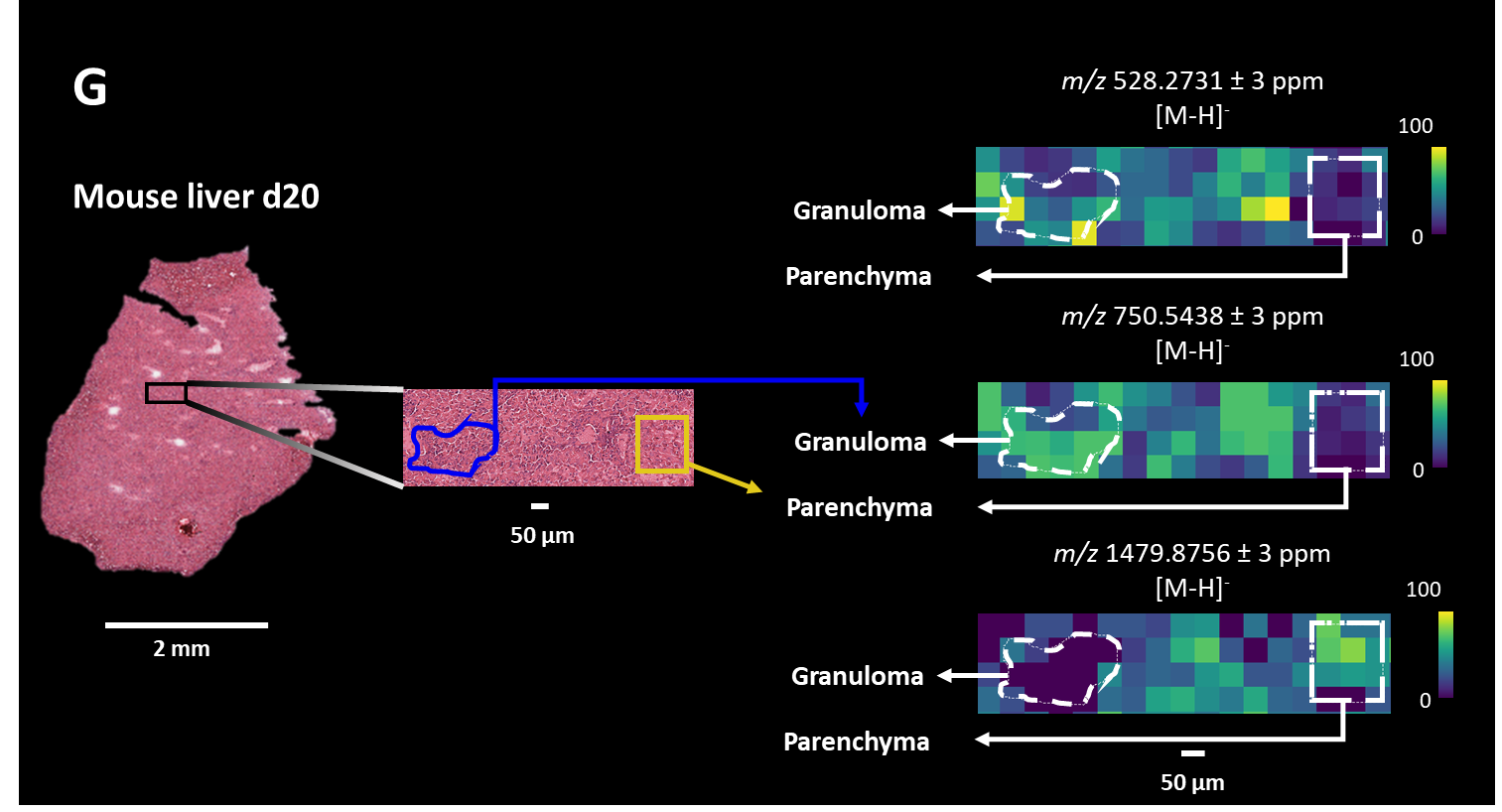


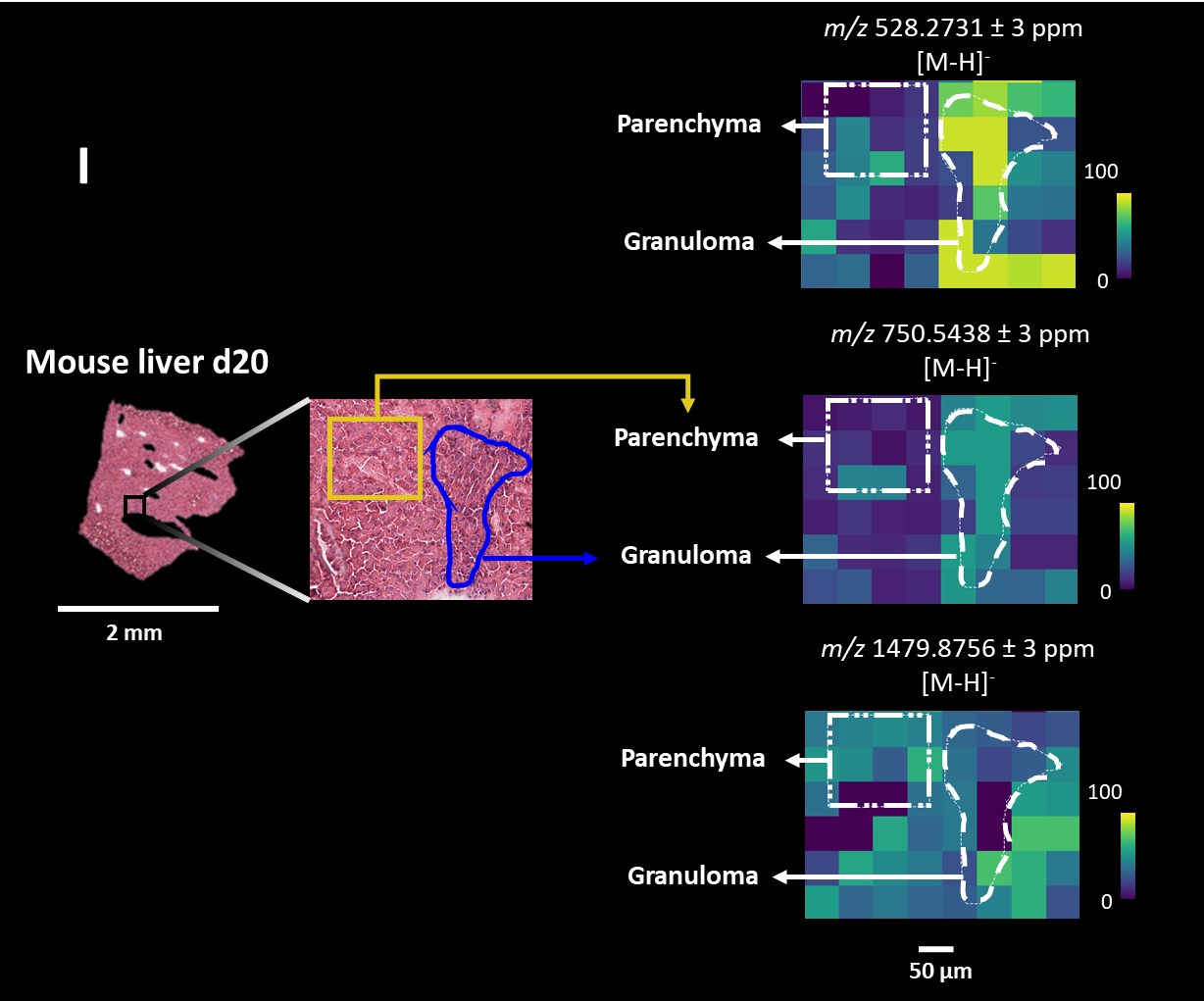


**Figure S6**: Pathology-guided mass spectrometry imaging workflow, which shows the ability to deduce analyzed lipid features from morphologic regions of interest (ROI): Zoomed-in histopathological characterization of granulomas (blue) and surrounding parenchyma (yellow) in a *L. donovani*-infected mice liver, 14 day post-infection (d14) (**B, D, F** and **H**) or in a *L. donovani*-infected mice liver, 20 day post-infection (d20) (**A, C, E, G** and **I**). Hematoxylin and eosin-stained images are co-registered with mass spectrometry images for subsequent spatial lipid analysis from annotated granuloma and parenchyma ROIs. Spatial distribution of *m/z* 528.2731 ± 3 ppm, *m/z* 750.5438 ± 3 ppm and 1479.8756 ± 3 ppm from the granuloma and parenchymal ROIs.


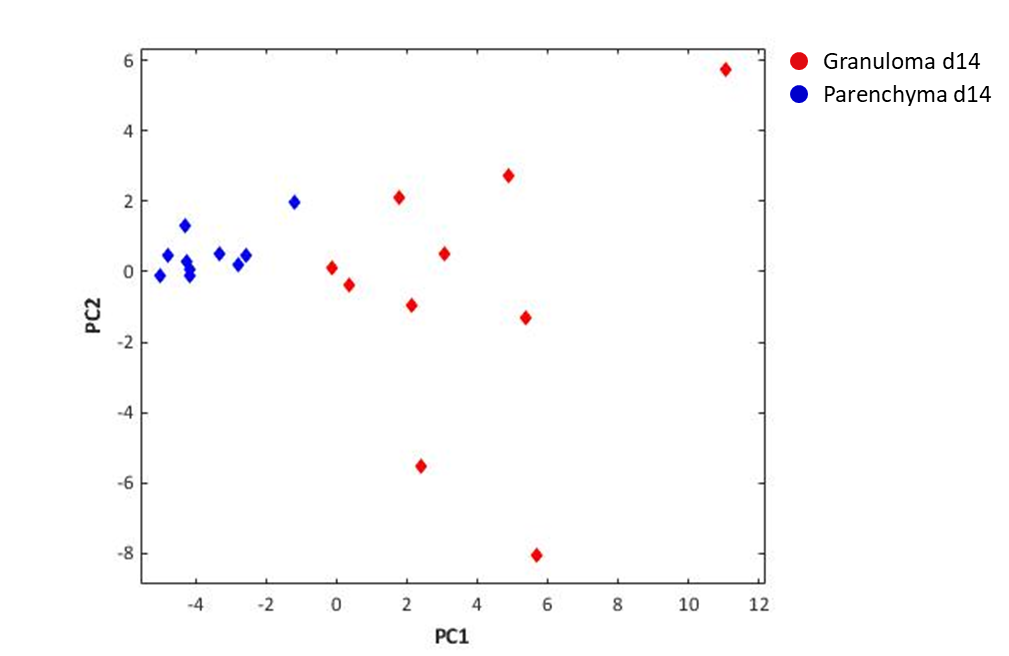


**Figure S7**: Principal component analysis (PCA) of granuloma and parenchymal ROIs from *Leishmania donovani*-infected mouse livers (d14).

**Table S2**: Log2 fold changes (F.C.) in average relative intensities of lipids identified between granulomas and parenchyma regions in both d14 (n=5) and d20 (n=5) infected mouse livers. Significance is defined as *p<0.05, **p<0.01 or ***p<0.001.

Phosphatidic acids (PA), phosphatidylethanolamines (PE), phosphocholine (PC) phosphatidylinositols (PI), phosphatidylserines (PS) and phosphatidylglycerols (PG).

| *m/z* [M - H]^-^ | Lipid ID | P-value | F.C. Granuloma d14 vs. Parenchyma d14 | F.C. Granuloma d20 vs. Parenchyma d20 |
| --- | --- | --- | --- | --- |
| 452.2782 | PE 16:0_0:0 | *** | 1.1 | -1.1 |
| 480.3095 | PE 18:0_0:0 | *** | 1.3 | 1.2 |
| 571.2887 | PI 16:0_0:0 | *** | 1.1 | -1.1 |
| 599.32 | PI 18:0_0:0 | * | 1.1 | 1.0 |
| 619.2887 | PI 20:4_0:0 | *** | 1.3 | 1.1 |
| 671.4655 | PA 16:0_18:2 | *** | -1.1 | -1.1 |
| 673.4811 | PA 16:0_18:1 | *** | 1.8 | 1.4 |
| 695.4655 | PA 18:2_18:2 | *** | -1.3 | -1.2 |
| 699.4967 | PA 18:0_18:2 | *** | 1.8 | 1.5 |
| 714.5076 | PE 16:0_18:2 | *** | -1.0 | -1.1 |
| 719.4654 | PA 16:0_22:6 | *** | -1.4 | -1.3 |
| 722.5126 | PE O-16:0_20:4 | *** | 3.4 | 2.1 |
| 723.4967 | PA 18:0_20:4 | *** | 1.4 | 1.3 |
| 738.5076 | PE 16:0_20:4 | *** | -1.1 | -1.1 |
| 742.5389 | PE 18:0_18:2 | *** | 1.2 | 1.1 |
| 747.4967 | PA 18:0_22:6 | *** | 1.3 | 1.2 |
| 750.5439 | PE O-18:0_20:4 | *** | 3.3 | 2.0 |
| 762.5076 | PE 16:0_22:6 | *** | -1.5 | -1.3 |
| 766.5388 | PE 18:0_20:4 | ** | 1.1 | 1.1 |
| 771.5179 | PG 18:1_18:2 | *** | 2.4 | 1.5 |
| 773.5332 | PG 18:0_18:2 | *** | 1.7 | 1.4 |
| 782.4974 | PS 16:0_20:4 | ** | 1.2 | -1.0 |
| 786.5286 | PS 18:0_18:2 | *** | 3.5 | 2.1 |
| 788.5233 | PE 18:1_22:6 | *** | 1.4 | 1.3 |
| 790.5388 | PE 18:0_22:6 | *** | -1.0 | -1.0 |
| 806.4973 | PS 16:0_22:6 | *** | -1.1 | -1.2 |
| 810.5286 | PS 18:0_20:4 | *** | 1.4 | 1.2 |
| 833.5181 | PI 16:0_18:2 | *** | 1.2 | 1.0 |
| 838.5598 | PS 18:0_22:4 | *** | 3.5 | 2.1 |
| 857.5181 | PI 16:0_20:4 | *** | -1.0 | -1.1 |
| 881.5181 | PI 16:0_22:6 | *** | 1.2 | 1.1 |
| 883.5338 | PI 18:1_20:4 | *** | 1.1 | -1.0 |
| 909.5492 | PI 18:0_22:6 | *** | 1.7 | 1.4 |
| 913.5805 | PI 18:0_22:4 | *** | 2.6 | 1.7 |

**Table S3**: Log2 fold changes (F.C.) in average relative intensities of lipids identified between granulomas in d14 (n=5) and d20 (n=5) and between surrounding parenchymal regions in d14 (n=5) and d20 (n=5) infected mouse livers. Significance is defined as *p<0.05, **p<0.01 or ***p<0.001.

Phosphatidic acids (PA), phosphatidylethanolamines (PE), phosphocholine (PC) phosphatidylinositols (PI), phosphatidylserines (PS) and phosphatidylglycerols (PG).

| *m/z* [M - H]^-^ | Lipid ID | P-value | F.C. Granuloma d14 vs. Granuloma d20 | F.C. Parenchyma d14 vs. Parenchyma d20 |
| --- | --- | --- | --- | --- |
| 452.2782 | PE 16:0_0:0 | *** | -1.1 | -1.3 |
| 480.3095 | PE 18:0_0:0 | *** | -1.1 | -1.2 |
| 571.2887 | PI 16:0_0:0 | *** | -1.2 | -1.4 |
| 599.32 | PI 18:0_0:0 | * | -1.0 | -1.1 |
| 619.2887 | PI 20:4_0:0 | *** | -1.1 | -1.2 |
| 671.4655 | PA 16:0_18:2 | *** | -1.3 | -1.2 |
| 673.4811 | PA 16:0_18:1 | *** | -1.4 | -1.8 |
| 695.4655 | PA 18:2_18:2 | *** | 1.1 | 1.1 |
| 699.4967 | PA 18:0_18:2 | *** | -1.3 | -1.5 |
| 714.5076 | PE 16:0_18:2 | *** | -1.4 | -1.5 |
| 719.4654 | PA 16:0_22:6 | *** | -1.1 | -1.0 |
| 722.5126 | PE O-16:0_20:4 | *** | -1.2 | -2.0 |
| 723.4967 | PA 18:0_20:4 | *** | -1.0 | -1.1 |
| 738.5076 | PE 16:0_20:4 | *** | -1.1 | -1.1 |
| 742.5389 | PE 18:0_18:2 | *** | -1.2 | -1.3 |
| 747.4967 | PA 18:0_22:6 | *** | -1.2 | -1.3 |
| 750.5439 | PE O-18:0_20:4 | *** | 1.0 | -1.6 |
| 762.5076 | PE 16:0_22:6 | *** | -1.1 | -1.0 |
| 766.5388 | PE 18:0_20:4 | ** | -1.0 | -1.1 |
| 771.5179 | PG 18:1_18:2 | *** | -1.4 | -2.2 |
| 773.5332 | PG 18:0_18:2 | *** | -1.2 | -1.5 |
| 782.4974 | PS 16:0_20:4 | ** | 1.0 | -1.2 |
| 786.5286 | PS 18:0_18:2 | *** | -1.4 | -2.3 |
| 788.5233 | PE 18:1_22:6 | *** | -1.1 | -1.1 |
| 790.5388 | PE 18:0_22:6 | *** | -1.2 | -1.1 |
| 806.4973 | PS 16:0_22:6 | *** | -1.2 | -1.3 |
| 810.5286 | PS 18:0_20:4 | *** | -1.0 | -1.1 |
| 833.5181 | PI 16:0_18:2 | *** | -1.4 | -1.6 |
| 838.5598 | PS 18:0_22:4 | *** | -1.2 | -1.9 |
| 857.5181 | PI 16:0_20:4 | *** | -1.2 | -1.2 |
| 881.5181 | PI 16:0_22:6 | *** | -1.2 | -1.4 |
| 883.5338 | PI 18:1_20:4 | *** | -1.0 | -1.1 |
| 909.5492 | PI 18:0_22:6 | *** | -1.1 | -1.3 |
| 913.5805 | PI 18:0_22:4 | *** | 1.0 | -1.5 |
